# Intramyocardial injected human umbilical cord-derived mesenchymal stem cells (HucMSCs) contribute to the recovery of cardiac function and the migration of CD4^+^ T cells into the infarcted heart via CCL5/CCR5 signaling

**DOI:** 10.1186/s13287-022-02914-z

**Published:** 2022-06-11

**Authors:** Jing Liu, Xiaoting Liang, Mimi Li, Fang Lin, Xiaoxue Ma, Yuanfeng Xin, Qingshu Meng, Rulin Zhuang, Qingliu Zhang, Wei Han, Ling Gao, Zhiying He, Xiaohui Zhou, Zhongmin Liu

**Affiliations:** 1 Research Center for Translational Medicine, Shanghai East Hospital, Tongji University School of Medicine, 150 Jimo Rd, Pudong, Shanghai, 200120 People’s Republic of China; 2 Department of Burn and Plastic Surgery, Beijing Children’s Hospital, Capital Medical University, National Center for Children’s Health, Beijing, 100045 People’s Republic of China; 3 Institute for Regenerative Medicine, Shanghai East Hospital, School of Life Sciences and Technology, Tongji University, Shanghai, 200120 People’s Republic of China; 4 Shanghai Heart Failure Research Center, Shanghai East Hospital, Tongji University School of Medicine, Shanghai, 200120 People’s Republic of China; 5 Department of Cardiovascular Surgery, Shanghai East Hospital, Tongji University School of Medicine, 150 Jimo Rd, Pudong, Shanghai, 200120 People’s Republic of China; 6 Department of Heart Failure, Shanghai East Hospital, Tongji University School of Medicine, Shanghai, 200120 People’s Republic of China; 7 Shanghai Institute of Stem Cell Research and Clinical Translation, Shanghai, 200120 People’s Republic of China; 8 Translational Medical Center for Stem Cell Therapy and Institute for Regenerative Medicine, Shanghai East Hospital, Tongji University School of Medicine, Shanghai, 200123 People’s Republic of China; 9 Shanghai Engineering Research Center of Stem Cells Translational Medicine, Shanghai, 200335 People’s Republic of China

**Keywords:** Human umbilical cord-derived mesenchymal stem cells, Intramyocardial injection, Myocardial infarction, CD4^+^ T cells, CCL5/CCR5

## Abstract

**Background:**

Human umbilical cord-derived mesenchymal stem cells (HucMSCs) have been recognized as a promising cell for treating myocardial infarction (MI). Inflammatory response post MI is critical in determining the cardiac function and subsequent adverse left ventricular remodeling. However, the local inflammatory effect of HucMSCs after intramyocardial injection in murine remains unclear.

**Methods:**

HucMSCs were cultured and transplanted into the mice after MI surgery. Cardiac function of mice were analyzed among MI-N.S, MI-HucMSC and MI-HucMSC-C–C Motif Chemokine receptor 5 (CCR5) antagonist groups, and angiogenesis, fibrosis and hypertrophy, and immune cells infiltration of murine hearts were evaluated between MI-N.S and MI-HucMSC groups. We detected the expression of inflammatory cytokines and their effects on CD4^+^ T cells migration*.*

**Results:**

HucMSCs treatment can significantly improve the cardiac function and some cells can survive at least 28 days after MI. Intramyocardial administration of HucMSCs also improved angiogenesis and alleviated cardiac fibrosis and hypertrophy. Moreover, we found the much higher numbers of CD4^+^ T cells and CD4^+^FoxP3^+^ regulatory T cells (Tregs) in the heart with HucMSCs than that with N.S treatment on day 7 post MI. In addition, the protein level of C–C Motif Chemokine Ligand 5 (CCL5) greatly increased in HucMSCs treated heart compared to MI-N.S group. In vitro, HucMSCs inhibited CD4^+^ T cells migration and addition of CCL5 antibody or CCR5 antagonist significantly reversed this effect. In vivo results further showed that addition of CCR5 antagonist can reduce the cardioprotective effect of HucMSCs administration on day 7 post MI injury.

**Conclusion:**

These findings indicated that HucMSCs contributed to cardiac functional recovery and attenuated cardiac remodeling post MI. Intramyocardial injection of HucMSCs upregulated the CD4^+^FoxP3^+^ Tregs and contributed to the migration of CD4^+^ T cells into the injured heart via CCL5/CCR5 pathway.

**Supplementary Information:**

The online version contains supplementary material available at 10.1186/s13287-022-02914-z.

## Highlights

- HucMSCs with intramyocardial injection can survive in murine myocardium for at least 28 days and improve cardiac function post MI.
- HucMSCs treatment contributed to the angiogenesis and attenuated cardiac fibrosis and hypertrophy in murine MI models.
- Intramyocardial administration of HucMSCs may upregulated the migration of CD4^+^ T cells (especially CD4^+^FoxP3^+^ Tregs) into the injured heart via CCL5/CCR5 pathway.

## Introduction

Cardiovascular diseases, with high prevalence, threaten human health and cause great economic burden. Myocardial infarction (MI) is a common cardiovascular disease, which refers to the severe and persistent ischemic necrosis of the myocardium after the coronary artery is interrupted [1]. Although current clinical treatments (such as percutaneous coronary intervention/PCI and coronary artery bypass graft/CABG) have some therapeutic effects, the disability and mortality of MI patients are still high due to extensive myocardial injury and poor prognosis [2]. Thus, it is essential to seek effective strategies to improve heart function and repair myocardium post ischemic injury.

Transplantation of mesenchymal stem cells (MSCs), representing one of the promising cell therapeutic strategies in MI, has attracted great attention recently [3]. Various sources of MSCs including those derived from bone marrow, adipose tissue, perinatal tissues, and pluripotent stem cells have been examined in animal models as well as in preclinical and clinical trials. Umbilical cord as a kind of perinatal tissue, can be an attainable MSCs source. Compared with adult tissue derived MSCs, human umbilical cord-derived MSCs (HucMSCs) have longer telomerase and activity, higher yield, shorter doubling time and relatively lower immunogenicity [4, 5]. Recent studies confirmed the protective function of HucMSCs in acute MI animal models either by immediate intracoronary delivery [6], intravenous injection [7] or direct intramyocardial injection [5]. In two clinical trials [8], delayed intracoronary HucMSCs transfer was used in reperfused acute MI. These preliminary results indicated the safety and efficacy of HucMSCs in acute MI.

Exaggerated or persistent inflammatory activation after MI leads to poor adaptive healing and subsequent left ventricular remodeling [9]. Accumulating evidences suggested that MSCs therapy can regulate the balance between inflammation and repair after MI [6, 10]. Intravenous delivery of human bone marrow-derived MSCs (BMSCs) demonstrated a systemic anti-inflammatory activity [10]. Recent study showed that intracoronary administration of HucMSCs regulate T cells mediated inflammation and reduce cardiac injury [6]. In the present study, we investigated how intramyocardial administration of HucMSCs regulate the immune response in the injured heart and contribute to cardiac healing in murine MI models.

## Materials and methods

### Ethic approval

All experiments involving mice were approved by the Institutional Animal Care and Use Committee of Tongji University (Shanghai, China; approval no: TJLAC-019-131). The human umbilical cords were obtained from full-term births and implemented following the approval of Ethic Committee and the informed consent of donors in Shanghai East Hospital.

### Cell culture

HucMSCs were isolated and cultured in good manufacturing practices (GMP) lab. In this experiment, the culture procedures of primary HucMSCs were as follows. The umbilical cord was placed in the 10 cm dish and cut into 2–3 cm tissue. Blood from the umbilical cord was washed and umbilical vein and artery were removed. The Wharton’s jelly was separated from the umbilical cord and cut into pieces (approximately 1 cm). Then, pieces of tissue were tiled and cultured in Minimum Essential Medium, Alpha (α-MEM, Corning, 10-022) supplemented with 5% UltraGRO-Advanced (Helios, HPCFDCGL50), and were incubated at 37 °C in a humidified atmosphere of 95% air and 5% CO_2_ until HucMSCs slowly crawled out of pieces. After that, the medium was changed every 2–3 day, and HucMSCs underwent passages using Trypin-Express (Gibco, 12,604,021) solution. HucMSCs were used at passage 4–5 (P4-5) for all experiments.

### Tumorigenicity evaluation of HucMSCs in vivo

The 6–8 weeks old male nude mice (BALB/c, 20–22 g) were purchased from SLAC Laboratory Animal Co., Ltd (Shanghai, China) and housed in the specific pathogen free (SPF) environment. The mice were bred at 20–25 °C at 12 h light/dark cycles, and given sterile water and food. To confirm the tumorigenicity of HucMSCs in vivo, nude mice were randomly (using computer-generated random numbers) divided into two groups named HucMSC and A549 groups (n = 7, per group), and injected subcutaneously into the left arm with 1*10^6^ HucMSCs or A549 cells, respectively. The injection of A549 (a lung cancer cell line) was used as the positive control group. The survival situation of each group was evaluated for 120 days.

### Multiple enzymes detection

After HucMSCs intramyocardial injection, blood (about 300 μL) from different groups was collected both on day 7 and 28, and the serum was isolated by centrifugation. Aspartate transaminase (AST), Alanine aminotransferase (ALT), Creatine (Cr), Lactate dehydrogenase (LDH), Creatine kinase (CK), Creatine kinase isoenzyme (CK-MB) levels were measured using Beckman AU680 (Beckman Coulter, Inc.) according to manufacturer’s instructions.

### Histological analysis

After euthanizing animals, mice tissues were fixed in 4% paraformaldehyde (PFA) overnight, embedded in paraffin and sectioned at 3–4 μm intervals. The sections were stained with Hematoxylin and eosin (H&E) and Masson’s Trichrome. For Masson’s Trichrome, fibrosis was quantified through the relative blue staining area of per heart compared the left ventricle surface, using Image J software (National Institutes of Health).

### MI model establishment and HucMSCs injection

The 8–10 weeks old male mice (C57BL/6 J; 24–28 g) were purchased from SLAC Laboratory Animal Co., Ltd (Shanghai, China) and housed in the SPF environment. The mice were bred at 20–25 °C at 12 h light/dark cycles, and given sterile water and food. In our study, we used the double-blinding method. On one hand, mice were randomly (using computer-generated random numbers) divided into different groups. On the other hand, the investigators were blinded to group allocation and assessed the outcome after difference groups were treated.

Before establishing MI models, HucMSCs were counted and resuspended in N.S at a final concentration of 3*10^5^ cells per mouse. Next, mice were anesthetized with sodium pentobarbital (60 mg/kg body weight, intraperitoneal injection), and the MI operation was started when the toes of the mice showed no reaction after extrusion. Then mice were intubated and mechanically ventilated using a rodent respirator (Shanghai Alcott Biotechnology Co., Ltd, ALC-V8S). A left thoracotomy at the fourth-fifth intercostal space was performed, and the left coronary artery was ligated. Then, mice were immediately injected 30 μL of 3*10^5^ HucMSCs or N.S by three points into the areas adjacent to the infarcted tissue (10 μL per point) with a 30-gauge needle gas-tight syringe (Hamilton Company). The animal temperature was maintained during surgery by using a heating pad. The convalescent mice after surgery were placed in a separate cage to avoid injury by the unoperated mice. After operation, all animals were monitored and received meloxicam (5 mg/kg) and buprenorphine (0.05 mg/kg) for pain management. Finally, the animals were euthanized after 7 or 28 days and tissues were collected for following experiments. For Inclusion/exclusion criteria, mice (8–10 weeks old, male) with normal and similar weigh (24-28 g), appearance and hair were included in the study. Before operation, the baseline cardiac function of each mouse was evaluated by echocardiography, and mice with LVEF below 50% were excluded from the study. During operation, we included the mice only when the ligation of LAD was successful (the anterior wall of left ventricle turned white).

### Echocardiography

Mice were anesthetized with 2% isoflurane inhalation and analyzed using a Vevo 2100 high-resolution imaging system with a 30-MHz linear transducer (FUJIFILM Visual-Sonics, Inc.) as previously described [11]. We acquired and analyzed the cardiac function of each group through long-axis scans using M-mode images including left ventricular ejection function (LVEF), left ventricular fractional shortening (LVFS), left ventricular end diastolic/systolic volume (LVEDV/LVESV) and left ventricular end diastolic/systolic diameter (LVEDD/LVESD) both on day 7 and 28.

### Fluorescent labeling of HucMSCs

HucMSCs at P3 were labeled with letivirus containing green fluorescence protein (GFP) at a multiplicity of infection of 10 as previously described [12]. Infection efficiency was confirmed by GFP fluorescent signal viewed under the microscope.

### Bioluminescence imaging in vivo

HucMSCs were pre-stained with the near-infrared fluorescent-lipophilic tracer 1,1-dioctadecyl-3,3,3,3-tetramethylindotricarbocyanine iodide (DiR, ThermoFisher, D12731) according to the manufacturer’s instructions, and then 3*10^5^ DiR-labeled HucMSCs were injected in the heart of mice after MI operation. The fluorescence intensity of multiple organs (including heart, lung, liver, kidney and spleen) was analyzed using the IVIS imaging instrument (Xenogen, USA) with the relevant channel.

### Polymerase chain reaction (PCR)

Genomic PCR for human Alu-sx repeat sequences in heart tissue was performed as previously reported [13]. The primer of human Alu-sx is: F:5’-GGCGCGGTGGCTCACG-3’, R:5’-TTTTTTGAGACGGAGTCTCGCTC-3’. The amplified products were determined by electrophoresis in 1.5% agarose gel supplemented with ethidium bromide.

### Immunofluorescence staining

Firstly, frozen sections of heart tissues were fixed with 4% PFA and blocked with 1% Bovine Serum Albumin (BSA) at 4 °C for 30 min. Then, the sections were incubated with wheat germ agglutinin (WGA, Thermo Fisher, W11261), anti-CD31 (Cell Signaling, 3528S), anti-CD3 (Abcam, ab231775), anti-CD4 (Abcam, ab183685), anti-FoxP3 (Abcam, ab215206), anti-Ki67 (Abcam, ab16667) and human mitochondrion antibodies (Abcam, ab92824), at 4 °C overnight to visualize cardiac cells, immune cells and HucMSCs in heart tissues, respectively, while 2-(4-Amidinophenyl)-6-indolecarbamidine dihydrochloride (DAPI) was used to mark cell nuclei. After incubated with above antibodies and washed with phosphate buffered saline (PBS), different fields of each slide were randomly selected and photograph under an inverted fluorescent microscope (Leica DM6000B, Germany).

### Real-time quantitative PCR (RT-qPCR)

According to the manufacturer’s instruction, the total RNA was extracted with TRIzol regent (Beyotime, R0016), and then the concentration of total RNA was quantified with NanoDrop2000 (Thermo Fisher Scientific, Inc.). cDNA was obtained by reverse transcription PCR using the PrimerScript RT reagent kit (Takara, RR0036A) at 37 °C for 15 min, and 85 °C for 5 s. The mRNA levels were detected by RT-qPCR with a SYBR Green Master Mix kit (Thermo Fisher, 4,385,617) using an ABI QuantStudio 6 Flex System. The mRNA levels were normalized to the endogenous control GAPDH. The relative gene quantification was calculated using the 2^−ΔΔCT^ method. Gene primers were listed in Table S1.

### Preparation of single cell and flow cytometry analysis

Briefly, mice were anesthetized with 1% sodium pentobarbital (60 mg/kg, intraperitoneal injection), and the heart was exposed and perfused with pre-cold PBS on day 7 after MI. Then, the heart was cut into 1 mm and put into the C-tube with collagenase II solution (1.5 mg/mL, added 500 × DNAse). During broken twice using gentleMACS™ Tissue Dissociators (Miltenyi Biotec, USA), the heart tissue was centrifuged at 37 °C for 30 min. After removing debris, the supernatant was filtered (70 μm strainer) to obtain single cell suspension. Cells were stained with the following labeled antibodies: anti-mouse CD45 (Biolegend, Clone I3/2.3), CD19 (Biolegend, Clone 1D3/CD19), CD3 (Biolegend, Clone 17A2), CD4 (Biolegend, Clone GK1.5), F4/80 (Biolegend, Clone BM8), Ly-6G (Biolegend, Clone 1A8). Data were acquired on a FACS Beckman flow cytometer (BD Biosciences, USA), and analysis was performed with Flow Jo software (BD Biosciences, USA).

### Cytokine profiling detection

For cytokine protein expression, the tissue samples of infarct and border area in heart of MI murine were collected and weighed. Then we added 500 μL ProcartaPlex Cell Lysis Buffer (EPX-99999-000) per 100 mg tissue. After tissue homogenization and centrifugation, the protein of sample was quantified by Bicinchoninic Acid (BCA) Protein Assay Kit (Bio-Rad, Hercules, CA, USA) and then detected for cytokine profiling (Thermo Fisher, EPX110-20820-901, EPX01A-20614-901, EPX01A-26005-901 and EPX01A-26009-901).

### The purification and culture of murine CD4^+^ T cells from spleen

First, RPMI 1640 complete medium (FBS 5 mL; PS 500 μL; HEPES buffer solution 500 μL; Nonessential Amino Acids 500 μL; Sodium Pyruvate solution 500 μL) and magnetic bead buffer (PBS 48.5 mL; FBS 1 mL; 1 mM EDTA 500 μL) were prepared. Next, the spleen was clipped and ground after the mice were anesthetized. Then, primary CD4^+^ T cells in the spleen were isolated by immunomagnetic bead (Thermo Fisher, 11415D). The density of CD4^+^ T cells was adjusted by cell count (1*10^6^ cells/per well) and planted to 24-well plate. CD4^+^ T cells were cultured with RPMI1640 complete medium and stimulated with anti-CD3/CD28 magnetic beads (cells:magnetic beads = 5:1, Gibco 00702195).

### Enzyme-linked immunosorbent assay (Elisa)

The concentrations of vascular endothelial growth factor (VEGF)-α in the tissue supernatants were measured using Quantikine Elisa kits for VEGF-α (Multi Sciences, EK283/2), according to the manufacturer’s instructions. All results were normalized to the total protein content.

### Cell migration assay

The heart tissues of normal mice were collected in control group, and the infarcted and border zone tissues of hearts were harvested in MI-N.S and MI-HucMSC groups. After the protein of cardiac tissues was lysed and quantified by BCA assay, the concentration of C–C Motif Chemokine Ligand 5 (CCL5) was quantified by Elisa assay (absin, abs520014) according to the manufacturer’s instructions. Polycarbonate membrane transwell inserts (24 well, pore size 5.0 μm) were used for CD4^+^ T cell migration assay. At the lower chamber, a total volume of 500 μL RPMI1640 containing 20 μg tissue protein were added. At the upper chamber, a total number 5*10^5^ purified CD4^+^ T cells were added. Sufficient dose CCL5 antibody (R&D system, MAB478) was added to the lower chamber for neutralization with information of CCL5 concentration determined by Elisa assay. Sufficient dose C–C Motif Chemokine receptor 5 (CCR5) antagonist (MCE, HY-13004) was added to the upper chamber with purified CD4^+^ T cells in indicated groups. After co-culturing for 16 h, the CD4^+^ T cells that had migrated to the lower chamber were collected and counted by handheld automated cell counter (millipore).

### Statistical analysis

The study was performed using GraphPad analysis software (version 8, USA), and data were expressed as mean ± Standard Error of Mean (SEM). Firstly, the normal distribution of all data was assessed by using Shapiro–Wilk normality test. Then, an unpaired two-tailed Student’s t-tests was used for differences between two groups, and one-way analysis of variance (ANOVA) followed by Bonferroni test was used for multiple comparisons. The survival curve was estimated by the log-rank test. *p* < 0.05 was considered statistically significant difference.

## Results

### The phenotype and safety evaluation of HucMSCs

Firstly, flow cytometry analysis demonstrated that HucMSCs express the typical surface markers of CD73, CD105 and CD90, while CD11b, CD19, CD34, CD45 and HLA-DR are absent in these cells (Additional file 1: Fig. S1A). Additional file 1: Fig. S1B showed the tri-lineage including osteogenic, adipogenic and chondrogenic differentiation potential of HucMSCs.

**Fig. 1:**
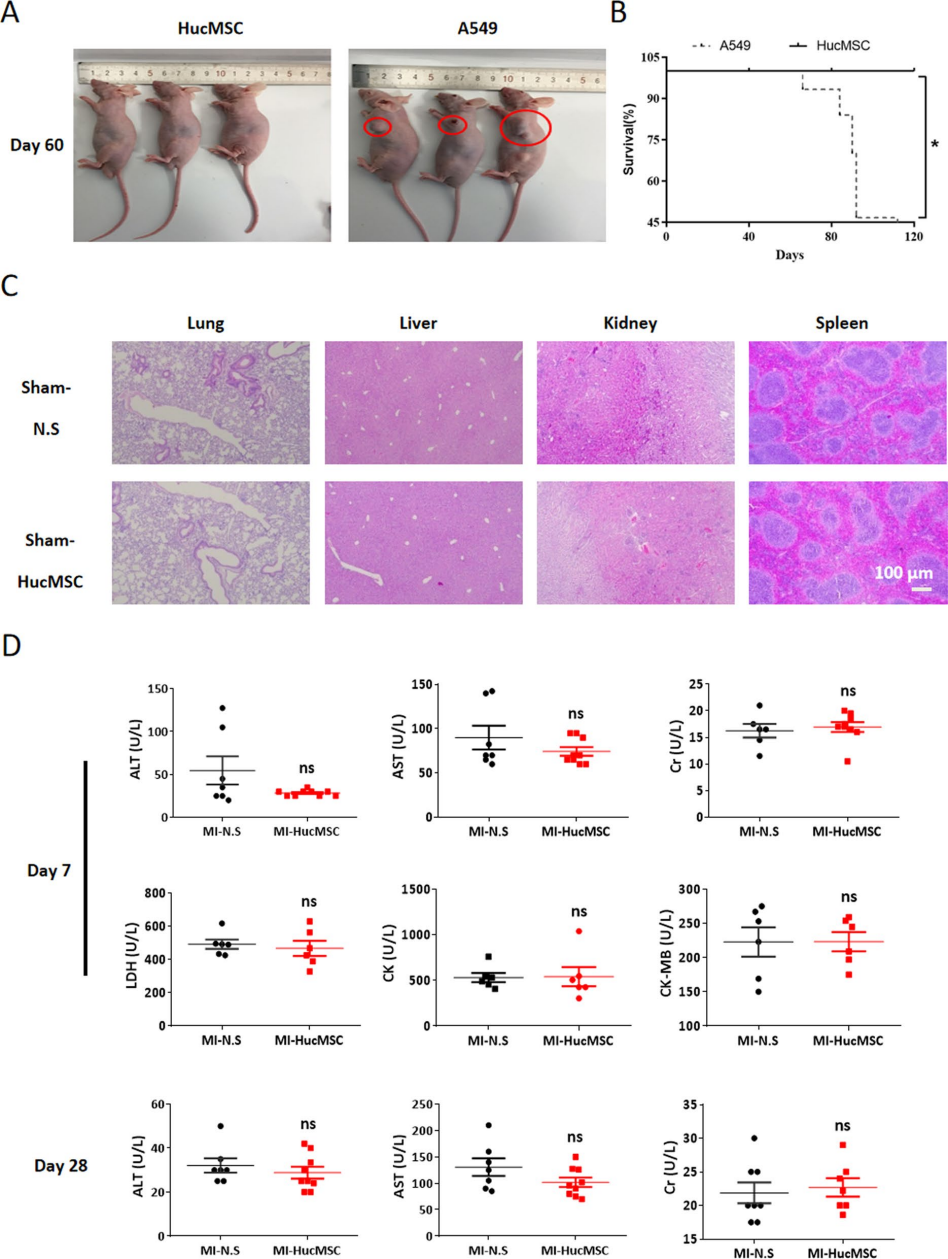
The safety evaluation of HucMSCs in vivo. **A** Representative images of subcutaneous tumor formation between HucMSC and A549 groups on day 60. **B** The survival curves of HucMSC and A549 groups were observed for 120 days. *p < 0.05 versus A549 group. **C** Representative H&E immunohistochemical photos of the lung, liver, kidney and spleen on day 28. Scale bar = 100 µm. **D** The AST, ALT, Cr, LDH, CK, CK-MB levels in serum were measured in different groups on day 7 or 28. ns, no significant difference versus MI-N.S group. MI, myocardiac infarct; N.S, normal saline; HucMSCs, Human umbilical cord-derived mesenchymal stem cells; AST, aspartate transaminase; ALT, alanine aminotransferase; Cr, Creatine; LDH, Lactate dehydrogenase, CK, Creatine kinase; CK-MB, Creatine kinase isoenzyme

Then, nude mice were applied to assess the safety of HucMSCs in vivo. After 1*10^6^ A549 cells injection, a subcutaneous tumor was occurred in the left arm of nude mouse on day 60 (Fig. 1A). But no tumor formation was observed and no mice died in the HucMSC group for consecutive 120 days compared with A549 group (Fig. 1B). H&E staining results showed that intramyocardial injection of HucMSCs had no obvious impact on the pathology of multiple organs, including the lung, liver, kidney and spleen on day 28 (Fig. 1C). Meanwhile, we didn’t found many local heart tissue disruptions in HucMSCs treated group on day 7 after H&E staining (Additional file 1: Fig. S2). In addition, intramyocardial treatment with HucMSCs did not affect the levels of ALT, AST, Cr in serum on day 7 and 28 nor affect LDH, CK and CK-MB on day 7 in murine MI models (Fig. 1D).

### Intramyocardial administration of HucMSCs improved cardiac function post MI

**Fig. 2:**
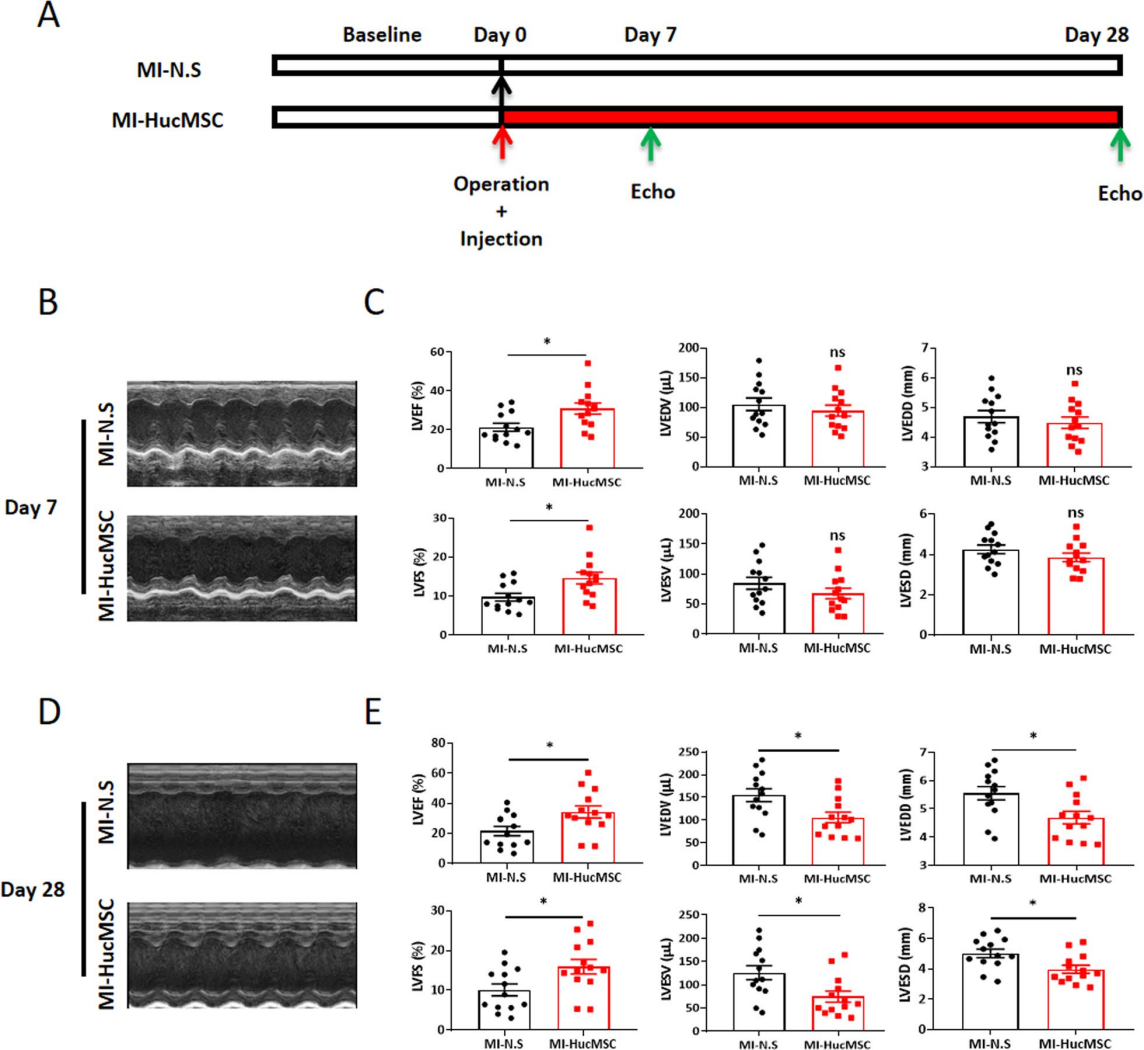
HucMSCs administration improved cardiac function of mice post MI injury. **A** The flow diagram of the experiment design. The two groups: MI-N.S and MI-HucMSC. After MI injury was established, mice were immediately injected N.S or HucMSCs by three points into the peri-infarcted heart zone. **B** Representative echocardiographic images (M-mode) in two groups on day 7 following MI surgery. **C** Statistical results of cardiac function on day 7 between MI-N.S and MI-HucMSC groups (n = 13 per group). **D** Representative echocardiographic images (M-mode) in two groups on day 28. **E** Statistical results of cardiac function on day 28 between MI-N.S and MI-HucMSC groups (n = 13 per group) following MI surgery. *p < 0.05 versus MI-N.S group. *LVEF* left ventricular ejection fraction; *LVFS* left ventricular fractional shortening; *LVEDV/LVESV* left ventricular end diastolic/systolic volume; *LVEDD/LVESD* left ventricular end diastolic/systolic diameter

To determine whether HucMSCs exerted a protective role in murine MI models, 3*10^5^ HucMSCs were injected in the peri-infarcted area. The flow chart of the experiment was shown in Fig. 2A, and cardiac function was tested by M-mode echocardiography both on day 7 and 28 after HucMSCs intramyocardial treatment. As shown by Fig. 2B–E, administration of HucMSCs significantly improved LVEF and LVFS both on day 7 and 28, and further reduced LVEDV/LVESV and LVEDD/LVESD on day 28 compared to the N.S treated MI mice.

### Retention of HucMSCs in vivo after intramyocardial injection

**Fig. 3:**
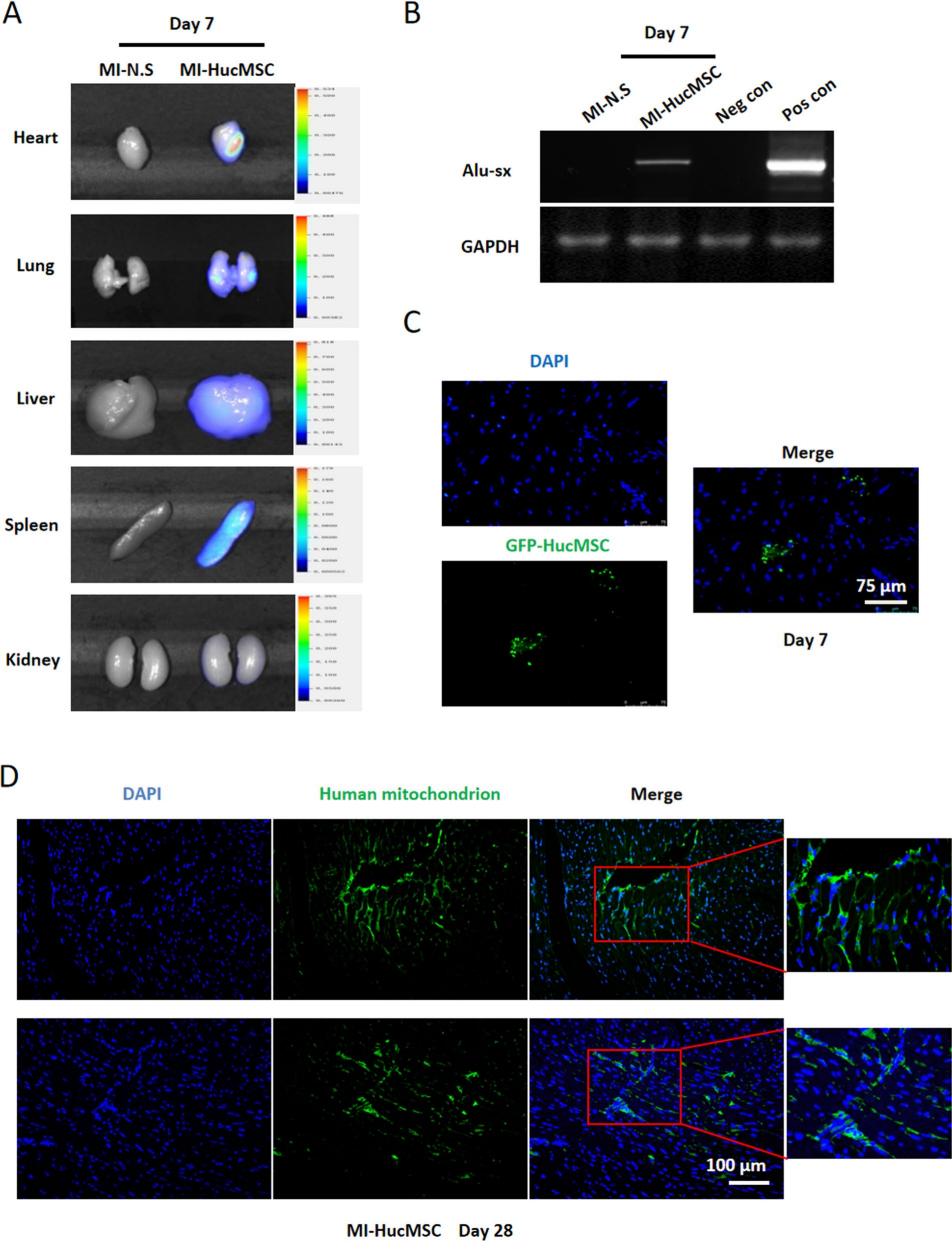
Retention of HucMSCs in vivo. **A** The fluorescence intensity of DiR-labeled HucMSCs were detected in multiple organs including heart, lung, liver, spleen and kidney on day 7 after MI. **B** Compared with MI-N.S group, Alu-sx was positively expressed in MI-HucMSC group by genomic PCR. *Neg con* negative control; *Pos con* positive control; *PCR* ploymerase chain reaction. **C** Representative immunofluorescence images of GFP-HucMSC hearts on day 7. *GFP* green fluorescence protein. **D** Representative immunofluorescence images of MI-HucMSC hearts stained with human mitochondrion on day 28. Green, human mitochondrion; Blue, DAPI. Scale bar = 100 µm

Next, we confirmed the retention of HucMSCs in vivo after MI. DiR-labeled HucMSCs were administrated into the myocardium, and high intensity of HucMSCs signaling were detected in heart, lung, liver and spleen in MI-HucMSC group on day 7, compared with MI-N.S group (Fig. 3A). Figure 3B showed that human Alu-sx repeat sequences were found at the infarcted and border areas of heart in MI-HucMSC group on day 7 by genomic PCR, compared with MI-N.S group. Additionally, fluorescence results showed that green fluorescent signals were present in the border zone of the heart on day 7 when GFP-labeled HucMSCs were injected into the myocardium (Fig. 3C). And the immunofluorescence staining of human mitochondrion further verified the presence of HucMSCs in the myocardium at 28 days post MI (Fig. 3D).

### HucMSCs treatment contributed to the myocardial angiogenesis but attenuated fibrosis and hypertrophy in murine MI models

**Fig. 4:**
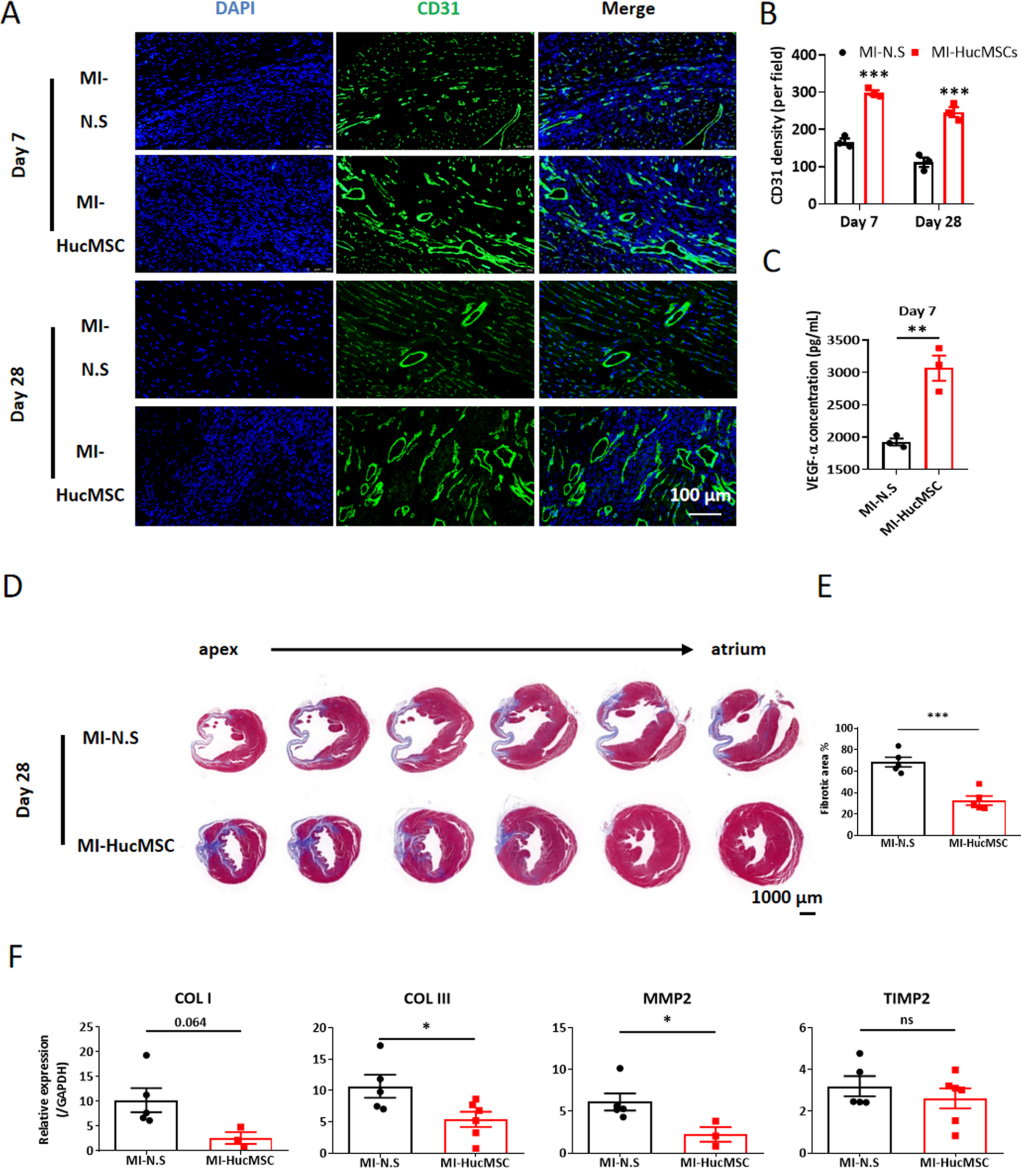

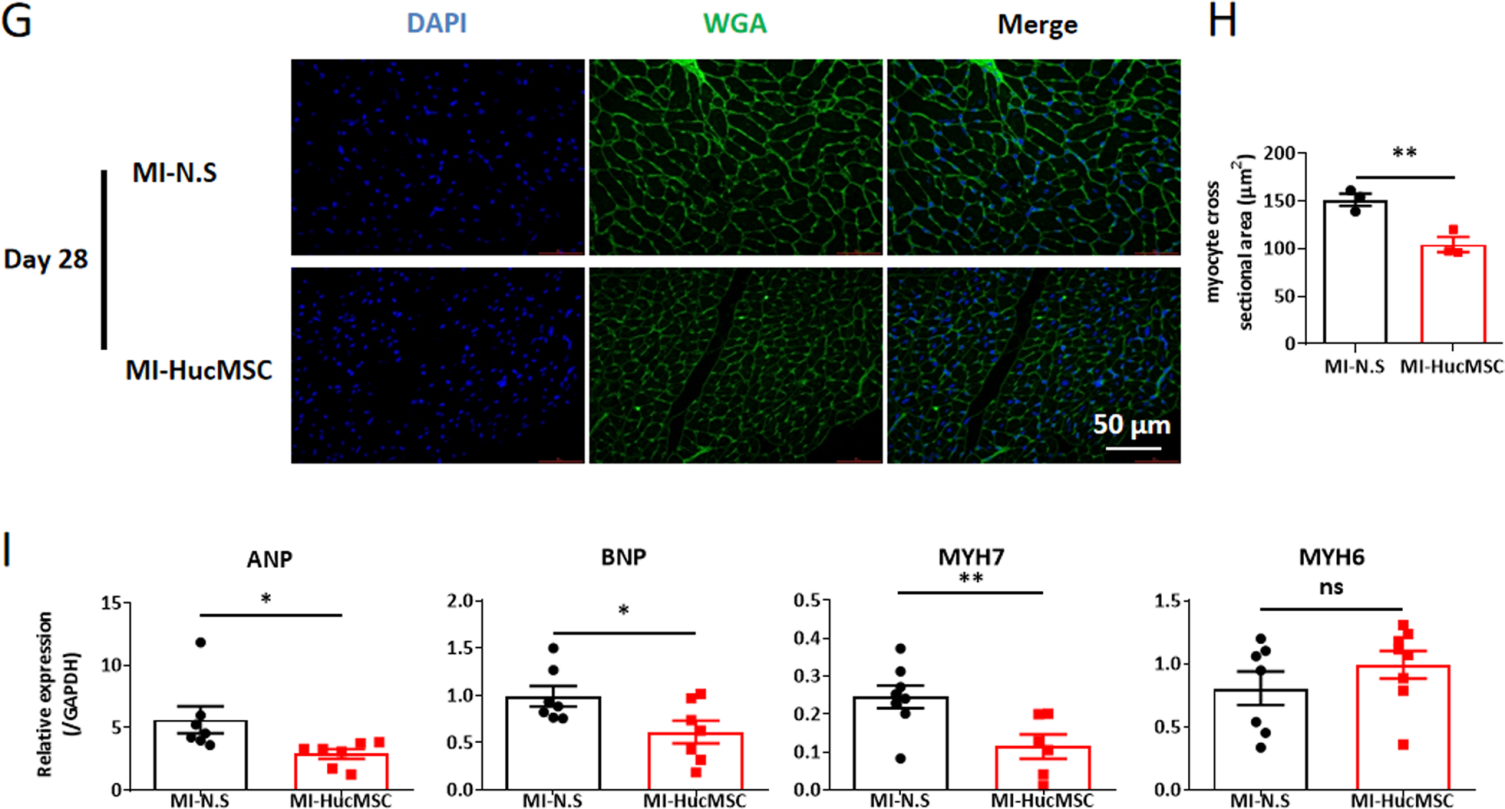
HucMSCs treatment contributed to the angiogenesis, attenuated myocardial fibrosis and hypertrophy in hearts after MI. **A** Representative immunofluorescence images of hearts stained with CD31 between MI-N.S and MI-HucMSC groups on both day 7 and 28. Green, CD31; Blue, DAPI. Scale bar = 100 µm. **B** Statistical results of the CD31 density (per field) of MI-N.S and MI-HucMSC groups both on day 7 and 28. **C** The mRNA expression of VEGF-α in infarcted hearts of two groups on day 7. **D** Representative Masson’s Trichrome-stained histological sections of hearts between MI-N.S and MI-HucMSC groups. Scale bar = 1000 µm. **E** The fibrosis area of heart tissues was measured using ImageJ software. **F** The mRNA levels of COL I, COL II, MMP-2 and TIMP2 in infarcted hearts of two groups. **G** Representative immunofluorescence images of hearts stained with WGA between MI-N.S and MI-HucMSC groups. Green, WGA; Blue, DAPI. Scale bar = 50 µm. **H** The cross-sectional areas of heart sections were estimated using ImageJ software. **I** The mRNA levels of ANP, BNP, MYH7 and MYH6 in infarcted hearts of two groups. *p < 0.05, **p < 0.01, ***p < 0.001 versus MI-N.S group. *VEGF* vascular endothelial growth factor; *COL* collage; *MMP* matrix metalloproteinase; *TIMP* tissue inhibitor of matrix metalloproteinase; *WGA* wheat germ agglutinin. *ANP* atrial natriuretic peptide; *BNP* brain natriuretic peptide, *MYH* myosin heavy chain

We further determined the role of HucMSCs on cardiac angiogenesis. As shown in Fig. 4A, B, compared to the MI-N.S group, the expression of endothelial marker CD31 was significantly increased at the peri-infarcted area in the MI-HucMSC group both on day 7 and 28. Moreover, HucMSCs treatment remarkably upregulated the protein level of vascular endothelial growth factor-α (VEGF-α) in the infarcted and border area of the heart on day 7 post MI (Fig. 4C).

In addition, HucMSCs treatment significantly alleviated the average area of myocardium fibrosis, as compared with MI-N.S group on day 28 (Fig. 4D, E). Meanwhile, compared to the MI-N.S heart, the expression of fibrosis markers including collagen III (COL III) and matrix metalloproteinase 2 (MMP2) significantly decreased in the MI heart treated with HucMSCs on day 28 (Fig. 4F). Moreover, the immunofluorescence staining of WGA (Fig. 4G, H) and the mRNA expression of hypertrophy markers (included atrial natriuretic peptide/ANP, brain natriuretic peptide/BNP, myosin heavy chain 7/MYH7) (Fig. 4I) together proved that HucMSCs administration attenuated cardiac hypertrophy on day 28 after MI.

### Increase of CD4^+^ T cells and CD4^+^FoxP3^+^ regulatory T cells in the infarcted heart after HucMSCs treatment

**Fig. 5:**
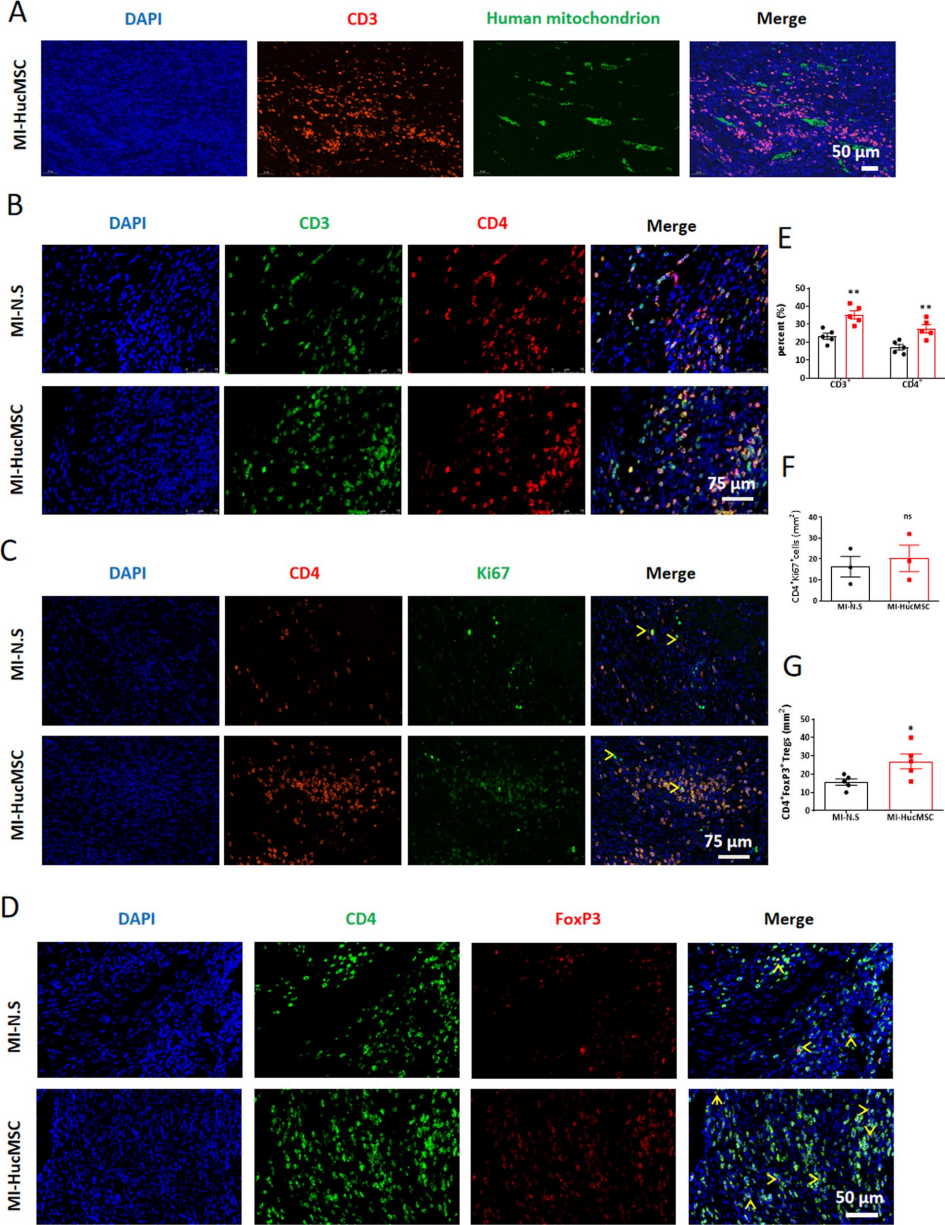

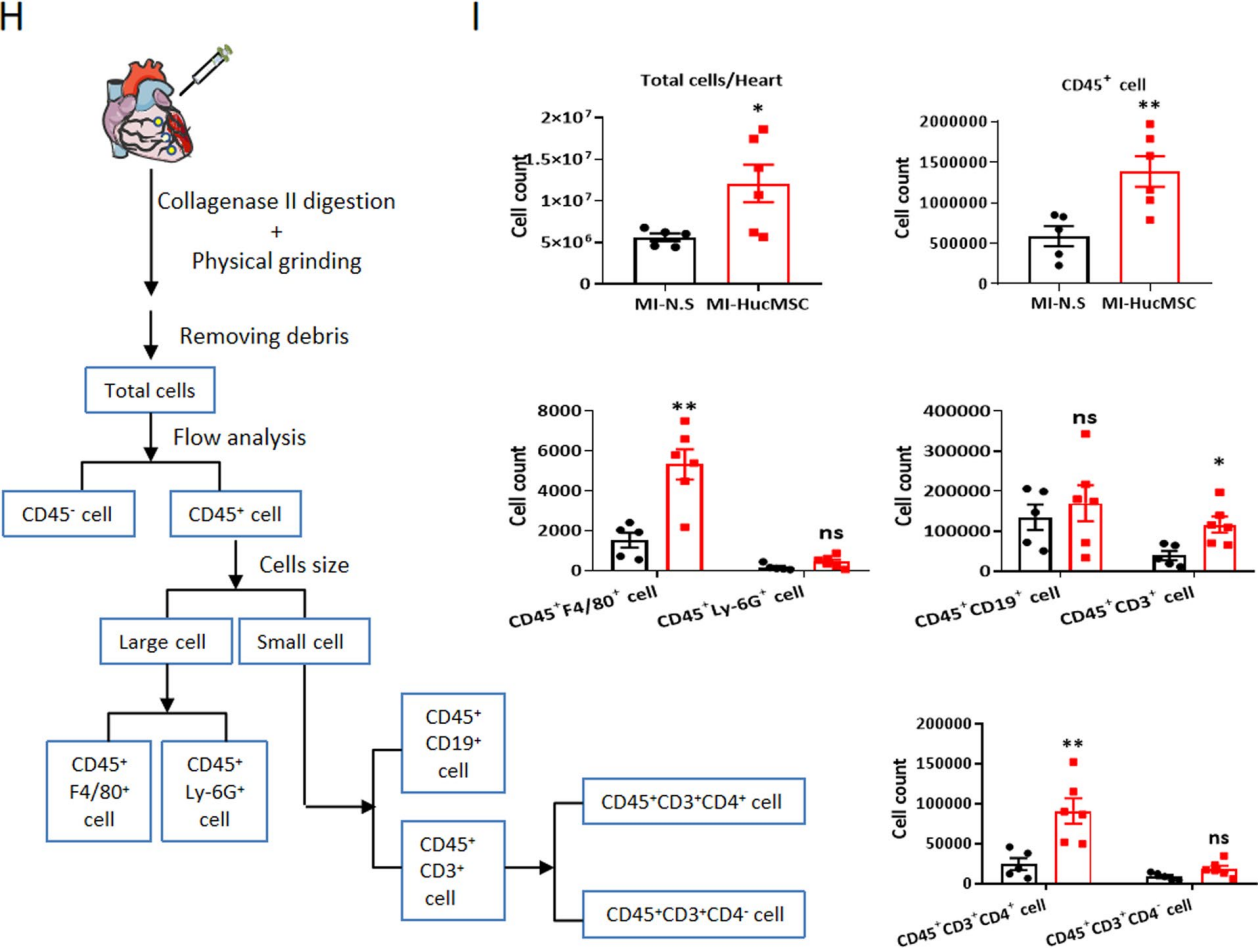
Increase of immune cells in the infarcted heart after HucMSCs treatment on day 7. **A** Representative immunofluorescence images of hearts stained with human mitochondrion and CD3/CD4 in MI-HucMSC groups. Green, HucMSCs; Red, CD3; Blue, DAPI. Scale bar = 50 µm. **B** Representative immunofluorescence images of hearts stained with CD3 and CD4 between MI-N.S and MI-HucMSC groups. Green, CD3; Red, CD4; Blue, DAPI. Scale bar = 75 µm. **C** Representative immunofluorescence images of hearts stained with CD4 and Ki67 between MI-N.S and MI-HucMSC groups. Red, CD4; Green, Ki67; Blue, DAPI. Yellow arrow indicates CD4^+^Ki67^+^ double positive cells. Scale bar = 75 µm. **D** Representative immunofluorescence images of hearts stained with CD4 and FoxP3 between MI-N.S and MI-HucMSC groups. Green, CD4; Red, FoxP3; Blue, DAPI. Yellow arrow indicates CD4^+^FoxP3^+^ double positive cells. Scale bar = 50 µm. **E** Statistical results of percent (%) of CD3^+^ or CD4^+^ T cells between MI-N.S and MI-HucMSC groups. **F** Statistical results of cell counts of CD4^+^Ki67^+^ T cells between MI-N.S and MI-HucMSC groups. **G** Statistical results of cell counts of CD4^+^FoxP3^+^ Tregs between MI-N.S and MI-HucMSC groups. **H** The flow diagram of the heart digestion and strategy of flow cytometry analysis. **I** Statistical results of cell counts of immune cells between MI-N.S and MI-HucMSC groups. *p < 0.05 and **p < 0.01 versus MI-N.S group

The inflammatory response after acute MI is critical in determining the infarcted size and subsequent adverse LV remodeling [14]. We next sought to verify whether administration of HucMSCs regulate the infiltration of immune cells into the heart on day 7. Firstly, large numbers of CD3^+^ T cells accumulated around the area of HucMSCs injection (Fig. 5A). Furthermore, compared to the N.S group, both percentages of CD3^+^ and CD4^+^ T cells increased around the peri-infarcted area of heart in HucMSCs treated group (Fig. 5B, E). But, Fig. 5C, F showed no significant difference in cell number of CD4^+^Ki67^+^ T cells on day 7 between these two groups. More importantly, the numbers of CD4^+^FoxP3^+^ regulatory T cells (Tregs) markedly upregulated in HucMSCs treated heart on day 7 (Fig. 5D, G) rather than day 28 (Additional file 1: Fig. S3).

At the same time, we digested the heart and analyzed the ratio and total numbers of immune cells in the heart (Fig. 5H). Compared with MI-N.S group, total numbers of single cells and CD45^+^ immune cells in the heart were increased in MI-HucMSC group through cell counts and calculating (total number multiply percentage) (Fig. 5I). Then the large size of CD45^+^ immune cells were gated to analyze macrophages (CD45^+^F4/80^+^) and neutrophils (CD45^+^Ly-6G^+^), while the small size of CD45^+^ immune cells were gated to analyze B cells (CD45^+^CD19^+^) and T cells (CD45^+^CD3^+^) (Fig. 5H). Flow cytometry results showed that the proportion of CD45^+^F4/80^+^ macrophage and CD45^+^CD3^+^ T cells in myocardial tissue were significantly enhanced in HucMSCs treatment compared with the N.S control on day 7 after MI (Additional file 1: Fig. S4). In particular, MI-HucMSC group significantly upregulated the percentage of CD45^+^CD3^+^CD4^+^ T cells but downregulated CD45^+^CD3^+^CD4^−^ T cells as compared with those from MI-N.S group on day 7 post MI (Additional file 1: Fig. S4). Similarly, the cell counts of CD45^+^F4/80^+^ macrophages, CD45^+^CD3^+^ T cells and CD45^+^CD3^+^CD4^+^ T cells in cardiac tissue were higher in HucMSCs than in N.S treated MI mice on day 7 by calculation (Fig. 5I).

### HucMSCs promoted CD4^+^ T cells migration into the injured heart via high levels of CCL5 post MI

**Fig. 6:**
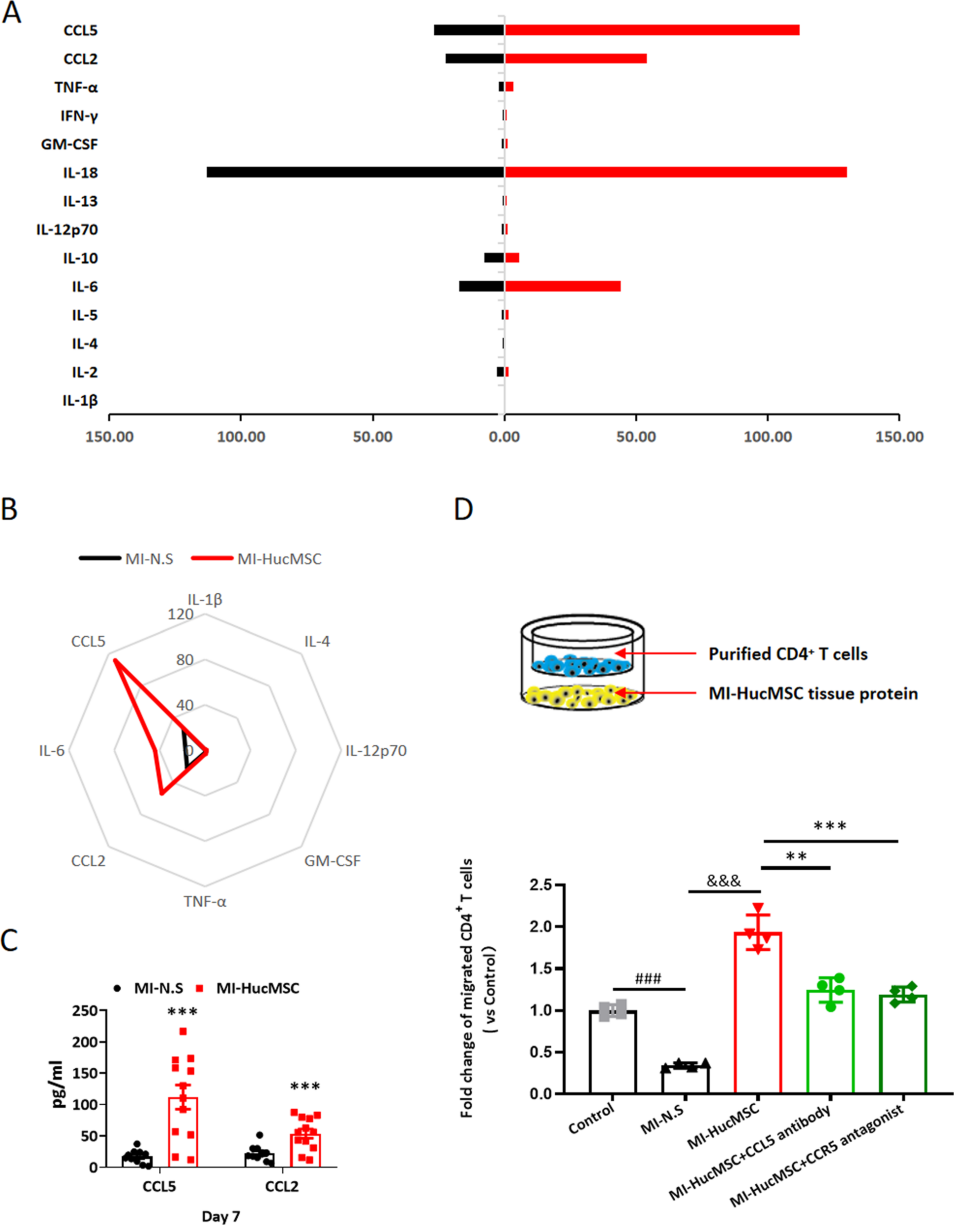
HucMSCs promoted CD4^+^ T cells migration into the injured heart via high levels of CCL5 post MI. **A** Statistical results of the T cells associated cytokines and chemokines in the heart between MI-N.S and MI-HucMSC groups on day 7. **B** The radar plots showed differences in cytokines between MI-N.S and MI-HucMSC groups on day 7. **C** The protein levels of CCL2 and CCL5 in heart tissues of two groups on day 7. *p < 0.05 and ***p < 0.001 versus MI-N.S group. **D** In vitro, statistical results of migrated CD4^+^ T cells number via different treatments. ^##^*p* < 0.01 versus Control group, ^&&&^*p* < 0.001 versus MI-N.S group, *p < 0.05 versus MI-HucMSC group

Then, we determined the mouse-specific cytokine profiling in the ischemic heart with different treatments. As shown in Additional file 1: Fig. S5 and Additional file 1: Fig. S6C, HucMSCs treatment significantly upregulated the protein levels of multiple cytokines and chemokines including IL-1β, IL-4, IL-6, IL-12P70, Granulocyte macrophage-Colony Stimulating Factor (GM-CSF), tumor necrosis factor-α (TNF-α), CCL2 and CCL5 on day 7 in infarcted myocardium compared with the N.S control. There were no statistical differences of IL-2, IL-5, IL-10, IL-13, IL-18 and Interferon-γ (IFN-γ) expressions between MI-N.S and MI-HucMSC groups (Additional file 1: Fig. S5). Among these molecules, CCL2 and CCL5 protein levels increased the most in the MI-HucMSC group (Fig. 6A, B).

To further verify whether CD4^+^ T cells infiltration in HucMSCs treated heart was mediated via CCL2 and CCL5, primary CD4^+^ T cells from spleen were applied and co-cultured with heart homogenate from MI-N.S or MI-HucMSC group in vitro. Figure 6D results confirmed that migrated cells number of CD4^+^ T cells significantly upregulated in MI-HucMSC group, compared with MI-N.S group. Addition of CCL5 antibody or CCR5 antagonist greatly suppressed the migration of CD4^+^ T cells induced by heart homogenate from MI-HucMSC group. Whereas neither CCL2 antibody nor CCR2 antagonist have significant effect on CD4^+^ T cells migration in vitro (Sup Fig. 6).

### The CCR5 antagonist inhibited the cardioprotective effect of HucMSCs administration post MI injury.

**Fig. 7:**
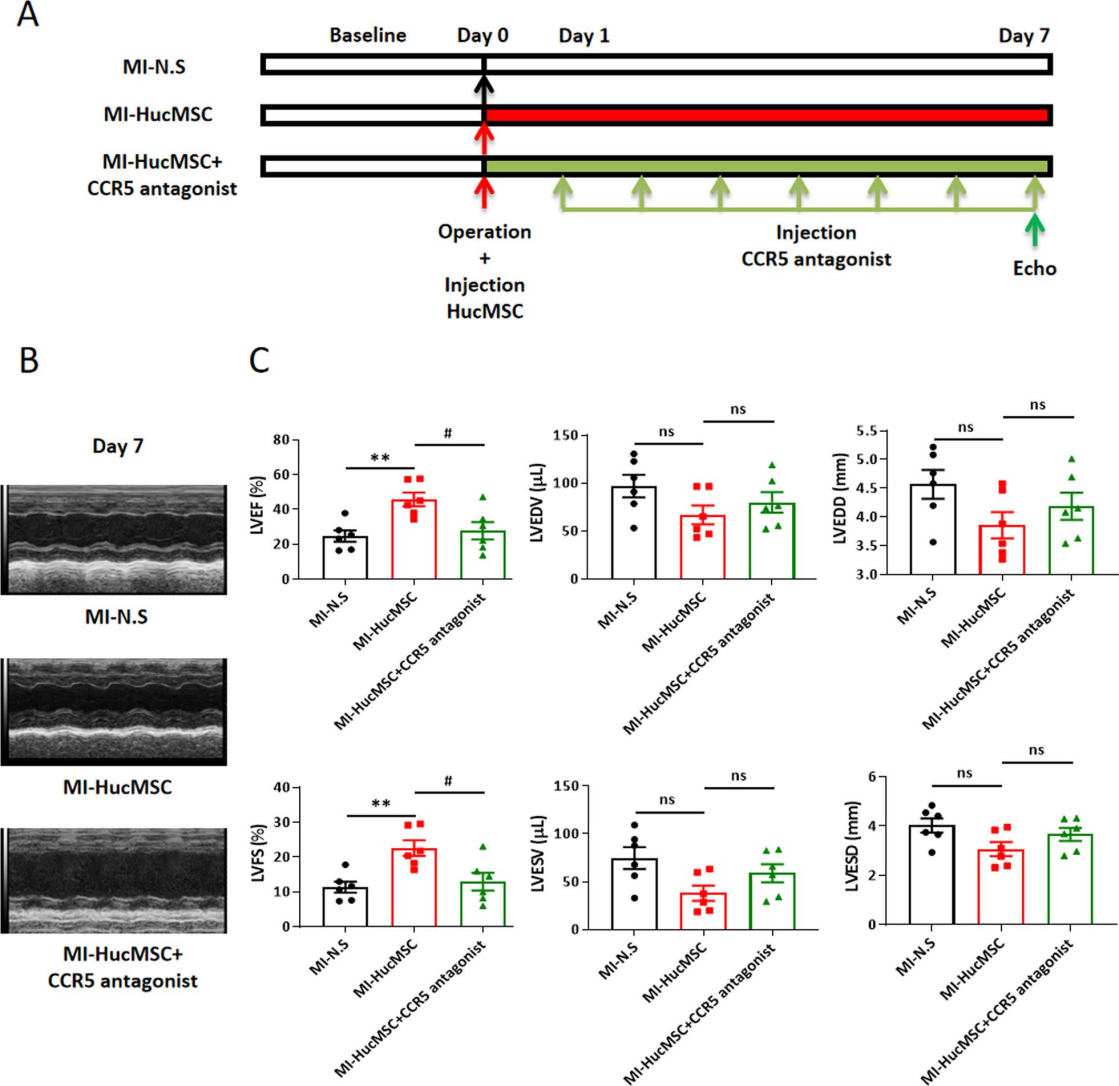
The CCR5 antagonist inhibited the cardioprotective effect of HucMSCs administration post MI injury. **A** The flow diagram of the experiment design. The three groups: MI-N.S, MI-HucMSC and MI-HucMSC-CCR5 antagonist. After MI injury was established, mice were immediately injected N.S or HucMSCs by three points into the peri-infarcted heart zone. Then, the MI-HucMSC-CCR5 antagonist group was intraperitoneally injected 20 mg/kg/d CCR5 antagonist. **B** Representative echocardiographic images (M-mode) in three groups on day 7 following MI surgery. **C** Statistical results of cardiac function on day 7 among MI-N.S (n = 6), MI-HucMSC (n = 6) and MI-HucMSC-CCR5 antagonist (n = 6) groups. *p < 0.05 versus MI-N.S group; ^#^*p* < 0.05 versus MI-HucMSC group

Thus, we performed the in vivo experiment by using CCR5 antagonist to determine the roles of CCL5/CCR5 in HucMSCs treatment mice. The flow chart of the experiment was shown in Fig. 7A, mice were intraperitoneally injected with 20 mg/kg/d CCR5 antagonist for 7 days, and then cardiac function was tested by M-mode echocardiography on day 7. As shown by Fig. 7B, C, administration of HucMSCs significantly improved LVEF and LVFS on day 7 compared to MI-N.S group, but administration of CCR5 antagonist reduced LVEF and LVFS on day 7 compared to MI-HucMSC group.

## Discussion

MSCs, as promising cell candidates for transplantation, have been widely used in various clinical diseases especially ischemic heart diseases [15]. Recent clinical study demonstrated the safety of intramyocardial transplantation of HucMSCs in patients subjected to chronic ischemic cardiomyopathy by monitoring the incidence of adverse events within the 12 months follow-up period [16]. In the present study, we firstly evaluated the safety of HucMSCs in vivo. We did not find subcutaneous tumor formation nor murine death till 120 days after HucMSCs injection. There were no pathological changes in organs till day 28 after intramyocardial injection of HucMSCs. In addition, intramyocardial administration of HucMSCs did not affect the expression of multiple enzymes nor cause local heart tissue disruptions. These results primarily confirmed the safety of intramyocardial injection of HucMSCs to mice.

Results from preclinical studies indicated the role of HucMSCs in improving the cardiac function in acute rat MI models [17]. Further randomized controlled trial suggested the significant efficacy of intramyocardial delivery of HucMSCs in chronic ischemic cardiomyopathy [16]. Consistent with these findings, intramyocardial administration of HucMSCs after MI can significantly improve LVEF and LVFS both on day 7 and 28, and further reduced LVEDV/LVESV and LVEDD/LVESD on day 28 in murine MI models.

Despite the efficacy, the utilization of MSCs is still challenged because of their low engraftment and survival rate in recipient hearts. In porcine model of MI, 6% of BMSCs by catheter-based transendocardial injection were tracked at 10 days [18]. Through positron emission tomography (PET) detection, only 1.3–2.6% BMSCs labeled with 18F-FDG were imaged after intracoronary injection at 50–75 min in MI patients [19]. Compared with intracoronary and interstitial retrograde coronary venous transplatation, intramyocardial injection had the most retention rate of BMSCs [20]. Our results further verified the presence of living HucMSCs after intramyocardial injection at least 28 days in murine infarcted myocardium.

Previous studies suggested the possible mechanisms by which HucMSCs improve cardiac function, including their transdifferentiation into cardiomyocyte-like cells by intramyocardial injection [21], the paracrine function contributing to angiogenesis, inflammation limitation, preservation of Cx43 gap junction by intravenous injection [7], and their regulation of fibrosis and apoptosis after intravenous administration [22]. Cardiomyocyte hypertrophy and interstitial fibrosis in the heart contribute to systolic and diastolic dysfunction, and ultimately to heart failure [23, 24]. In the present study, we found that intramyocardial administration of HucMSCs significantly promote cardiac angiogenesis and attenuated cardiac hypertrophy and fibrosis compared to MI-N.S group on day 28 after MI. The improvement may partly explain the efficacy of HucMSCs on chronic cardiac dysfuntion on day 28.

Acute MI is characterized by high levels of inflammatory responses, therefore, cardiac inflammation was reported to be an important therapeutic target [25]. Previous studies confirmed that activation and infiltration of inflammatory cells, especially CD4^+^ T cells, are closely related to myocardial repair after MI in a mouse model [25, 26]. In the present study, we found the adjacent locations of CD3^+^ T cells and HucMSCs in the border zone of the infarction. Further results revealed that both CD45^+^CD3^+^ T cells and CD45^+^CD3^+^CD4^+^ T cells increased in injury hearts after intramyocardial HucMSCs transplantation on day 7 after MI. While there is no significant difference of CD4^+^Ki67^+^ T cells in the heart between MI-HucMSC and MI-N.S group. These results suggested that HucMSCs treatment contribute to the infiltration, but not the proliferation of CD4^+^ T cells in the heart post MI. Tregs, an important subtype of CD4^+^ T cells known as an immune brake, can block excessive inflammatory responses and tissue destruction to maintain the homeostasis in vivo. Less recruitment of Tregs is associated with deteriorated LV dilation and increased expression of inflammatory mediators in the infarct zone in murine models [27]. Previous studies showed that Tregs were mainly recruited from the peripheral circulation to the heart after MI, and therapeutic activation or adoptive transfer of FoxP3^+^CD4^+^ Tregs can improve cardiac healing after MI [9, 28]. Intracoronary delivery of HucMSCs attenuate myocardial injury by promoting the generation of CD4^+^CD25^+^FoxP3^+^ Tregs [6]. Our results demonstrated that intramyocardial HucMSCs transplantation increased the CD4^+^FoxP3^+^ Tregs in the injured heart. Therefore, intramyocardial injection HucMSCs may improve the cardiac function mainly through increasing the infiltration of CD4^+^ T cells as well as CD4^+^FoxP3^+^ Tregs in the injured heart. Additionally, a study showed that HucMSCs sheet can also modulate macrophages mediated inflammation and preserve the cardiomyocytes from acute injury [29]. Recent reports showed that human MSCs or MSCs derived exsomes, may provide clinical strategies for wound healing and inflammatory disorders through significantly increasing M2 macrophage polarization from M1 macrophage [30, 31]. In the present study, we found that the level of CD45^+^F4/80^+^ macrophages increased in injury hearts after intramyocardial HucMSCs transplantation on day 7 after MI. Thus, macrophages may also play some roles in the immunomodulatory effect mediated by HucMSCs, and whether intramyocardial HucMSCs transplantation contribute to the differentiation of cardiac macrophages need further exploration.

In comparison with lymphoid organs, the preferential aggregation of Tregs in the injured hearts may depend on their upregulated expression of chemokines and chemokine receptors. Next, we explored which inflammatory cytokines signaling are involved in the recovery process of cardiac function and the migration of CD4^+^ T cells into the injured heart induced by HucMSCs. In our report, the cytokine profile indicated high levels of CCL2 and CCL5 in HucMSCs treated heart in vivo. The in vitro migration results further confirmed the inhibition of CCL5 or CCR5, rather than CCL2 or CCR2, significantly reversed the up-regulation of the migrated cell number of CD4^+^ T cells induced by heart homogenate in MI-HucMSC group. And our in vivo results further showed that addition of CCR5 antagonist can reduce the cardioprotective effect of HucMSCs administration on day 7 post MI injury. RANTES/CCL5 is secreted by endothelial cells, smooth muscle cells, macrophages and platelets, and it can induce leucocytes recruitment and activate T cells [32]. Previous reference reported that CCL5, secreted from macrophages, play an immunosuppressive role and the CCL5-CCR5 axis can home Tregs to injured area [33]. Destruction of the CCR5 leads to adverse remodeling and cardiac deterioration, which is associated with decreased infiltration of Tregs [34]. Adipose derived-MSCs can upregulate the secretion of IL-8 and CCL5 to promote T lymphocytes recruitment when exposed to inflammatory environment [35]. The present results suggested that intramyocardial HucMSCs treatment may induced the local cells of mouse to produce more CCL5 which promote the migration of CD4^+^ T cells and CD4^+^FoxP3^+^ Tregs into the injured heart.

In the present study, there are still some possible limitations as follow: Firstly, mice are widely used in the process to study diseases and explore mechanisms due to the low cost, rapid reproduction, small size and easy operation. Smaller animals (such as mice and rats) have much faster heart rates than larger animals (such as dogs or primates), more blood is pumped to their cells to provide the oxygen and nutrients. Faster heart rates will lead to lower rate of implantation and retention. Therefore, further studies were needed to translate the current small animal investigations to primates animals and clinical experiments. Secondly, although the direct injection of cells can successfully mend small damaged areas, transplantation by the needle injection of a cell suspension easily causes aggregation and necrosis of the grafted cells, and the shape, size, and location of transplanted cells are difficult to control, which can lead to lower survival rates, marginal engraftment, and suboptimal outcomes [36]. Thus, other HucMSCs delivery routes such as HucMSCs sheet or medium will further be assessed the retention and efficacy of HucMSCs on the cardiac function in murine MI models.

**Fig. 8:**
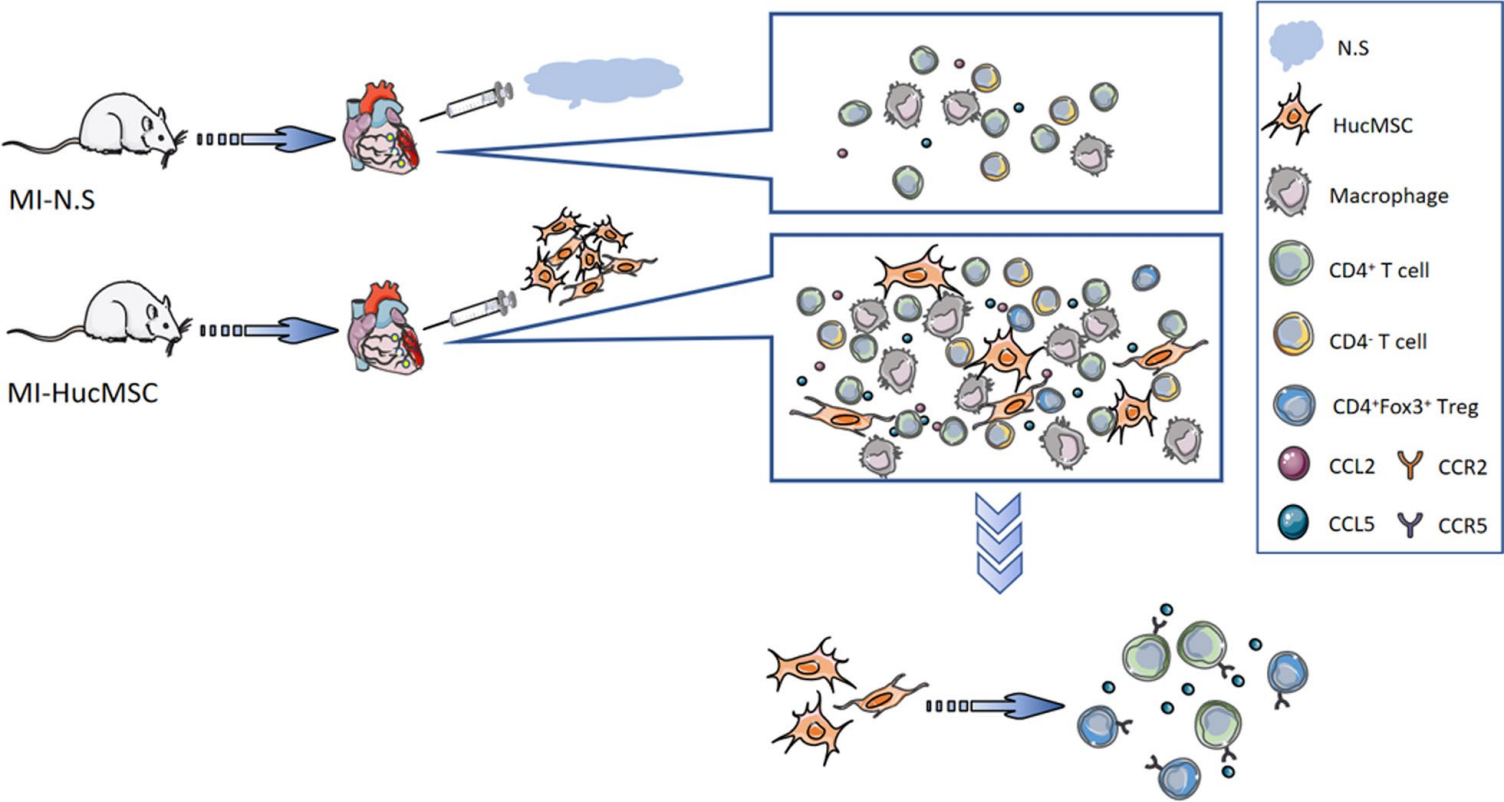
Schematic diagram of the role of HucMSCs in MI. Intramyocardial injection of HucMSCs upregulated the CD4^+^FoxP3^+^ Tregs and contributed to the migration of CD4^+^ T cells into the injured heart via CCL5/CCR5 pathway. *MI* myocardiac infarct; *N.S* normal saline; *HucMSCs* Human umbilical cord-derived mesenchymal stem cells; *Tregs* regulatory T cells; *CCL* C–C Motif Chemokine Ligand; *CCR* C–C Motif Chemokine receptor

Collectively, our animal study demonstrated that intramyocardial HucMSCs transplantation may represent a potential therapeutic strategy to improve the function in mice subjected to MI. We further confirmed that intramyocardial administration of HucMSCs upregulated the migration of CD4^+^ T cells (especially CD4^+^FoxP3^+^ Tregs) into the injured heart via CCL5/CCR5 pathway (Fig. 8). Therefore, further studies to evaluated the efficacy of targeting CCL5/CCR5 pathway in MI treatment are needed.

## Conclusion

This study indicated that HucMSCs with intramyocardial injection can survive in murine myocardium post MI at least 28 days. HucMSCs contributed to cardiac functional recovery and attenuated cardiac remodeling post MI. Intramyocardial injection of HucMSCs upregulated the CD4^+^FoxP3^+^ Tregs and contributed to the migration of CD4^+^ T cells into the injured heart via CCL5/CCR5 pathway.

## Supplementary Information

**Additional file 1:** Supplemental materials, methods, figures and tables.

## Data Availability

The data that support the findings of this study are available on request from the corresponding author.

## Abbreviations

MI: Myocardiac infarct
HucMSCs: Human umbilical cord-derived mesenchymal stem cells
SEM: Standard Error of Mean
AST: Aspartate transaminase
ALT: Alanine aminotransferase
Cr: Creatine
LDH: Lactate dehydrogenase
CK: Creatine kinase
CK-MB: Creatine kinase isoenzyme
LVEF: Left ventricular ejection fraction
LVFS: Left ventricular fractional shortening
LVEDV/LVESV: Left ventricular end diastolic/systolic volume
LVEDD/LVESD: Left ventricular end diastolic/systolic diameter
GFP: Green fluorescence protein
VEGF: Vascular endothelial growth factor
COL: Collage
MMP: Matrix metalloproteinase
TIMP: Tissue inhibitor of matrix metalloproteinase
WGA: Wheat germ agglutinin
ANP: Atrial natriuretic peptide
BNP: Brain natriuretic peptide
MYH: Myosin heavy chain
Tregs: Regulatory T cells
CCL: C–C Motif Chemokine Ligand
CCR: C–C Motif Chemokine Receptor
