# Correction: Intramyocardial injected human umbilical cord-derived mesenchymal stem cells (HucMSCs) contribute to the recovery of cardiac function and the migration of CD4^+^ T cells into the infarcted heart via CCL5/CCR5 signaling

**DOI:** 10.1186/s13287-025-04805-5

**Published:** 2025-11-18

**Authors:** Jing Liu, Xiaoting Liang, Mimi Li, Fang Lin, Xiaoxue Ma, Yuanfeng Xin, Qingshu Meng, Rulin Zhuang, Qingliu Zhang, Wei Han, Ling Gao, Zhiying He, Xiaohui Zhou, Zhongmin Liu

**Affiliations:** 1 Research Center for Translational Medicine, Shanghai East Hospital, Tongji University School of Medicine, 150 Jimo Rd, Pudong, 200120 Shanghai People’s Republic of China; 2 Department of Burn and Plastic Surgery, Beijing Children’s Hospital, National Center for Children’s Health, Capital Medical University, Beijing, 100045 People’s Republic of China; 3 Institute for Regenerative MedicineShanghai East Hospital, School of Life Sciences and Technology, Tongji University, Shanghai, 200120 People’s Republic of China; 4 Shanghai Heart Failure Research Center, Shanghai East Hospital, Tongji University School of Medicine, Shanghai, 200120 People’s Republic of China; 5 Department of Cardiovascular Surgery, Shanghai East Hospital, Tongji University School of Medicine, 150 Jimo Rd, Pudong, 200120 Shanghai People’s Republic of China; 6 Department of Heart Failure, Shanghai East Hospital, Tongji University School of Medicine, Shanghai, 200120 People’s Republic of China; 7 Shanghai Institute of Stem Cell Research and Clinical Translation, Shanghai, 200120 People’s Republic of China; 8 Translational Medical Center for Stem Cell Therapy and Institute for Regenerative Medicine, Shanghai East Hospital, Tongji University School of Medicine, Shanghai, 200123 People’s Republic of China; 9 Shanghai Engineering Research Center of Stem Cells Translational Medicine, Shanghai, 200335 People’s Republic of China

**Correction: Stem Cell Research & Therapy (2022) 13:247** 10.1186/s13287-022-02914-z

Following the publication of the article [1], the authors regretfully found one error in Fig. 1C which was intended to demonstrate the in vivo safety evaluation of HucMSCs. The selection error of the representative image of lung in Fig. 1C occurred during figure preparation.

The authors corrected the mistake and replaced the erroneous image with the correct one which is displayed ahead in this Correction article as the revised Fig. 1C. This unintentional error does not affect the conclusions of this study.


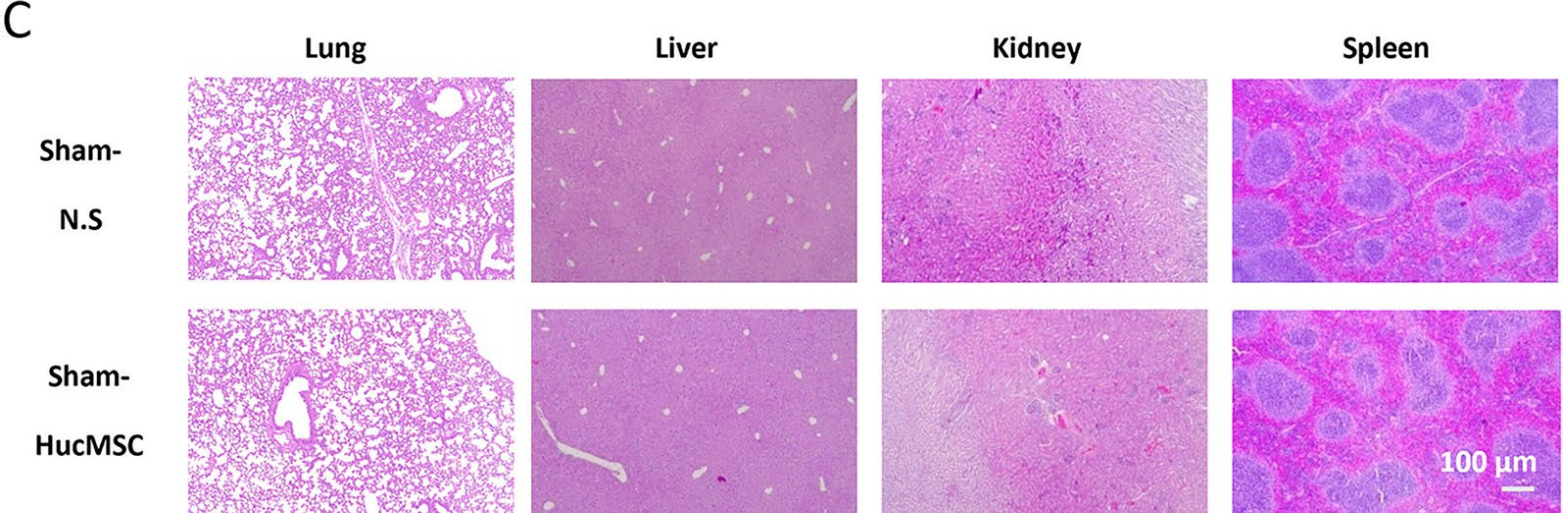


## References

[1] Liu J, et al. Intramyocardial injected human umbilical cord-derived mesenchymal stem cells (HucMSCs) contribute to the recovery of cardiac function and the migration of CD4 ^ +^T cells into the infarcted heart via CCL5/CCR5 signaling. Stem Cell Res Ther. 2022;13:247. 10.1186/s13287-022-02914-z. 35690805 10.1186/s13287-022-02914-z PMC9188247

