# Supplementary material for: Intramyocardial injected human umbilical cord-derived mesenchymal stem cells (HucMSCs) contribute to the recovery of cardiac function and the migration of CD4+ T cells into the infarcted heart via CCL5/CCR5 signaling
Source: Stem Cell Res Ther. 2022 Jun 11;13:247. doi: 10.1186/s13287-022-02914-z (PMC9188247; doi:10.1186/s13287-022-02914-z)
Supplement: Supplementary file 1 — Additional file 1: Supplemental materials, methods, figures and tables. [file 13287_2022_2914_MOESM1_ESM.docx]

**Supplemental material**

**Intramyocardial injected human umbilical cord-derived** **mesenchymal stem cells (HucMSCs) contribute to the recovery of cardiac function and the migration of CD4^+^ T cells into the infarcted heart via CCL5/CCR5 signaling**

Jing Liu^†1,2^, Xiaoting Liang^†1,3^, Mimi Li^1,4^, Fang Lin^1,4^, Xiaoxue Ma^1,4^, Yuanfeng Xin^1,5^, Qingshu Meng^1,4^, Rulin Zhuang^1,5^, Qingliu Zhang^6,7^, Wei Han^6,7^, Ling Gao^8^, Zhiying He^3,7,9^, Xiaohui Zhou^*1,4^, Zhongmin Liu^*1,4,5,7^

^†^Dr. Liu and Dr. Liang contributed equally to this work.

**Materials and methods**

**Cell culture**

HucMSCs were isolated and cultured in good manufacturing practices (GMP) lab. In this experiment, the culture procedures of primary HucMSCs were as follows. The umbilical cord was placed in the 10 cm dish and cut into 2-3 cm tissue. Blood from the umbilical cord was washed and umbilical vein and artery were removed. The Wharton’s jelly was separated from the umbilical cord and cut into pieces (approximately 1 cm). Then, pieces of tissue were tiled and cultured in Minimum Essential Medium, Alpha (α-MEM, Corning, 10-022) supplemented with 5% UltraGRO-Advanced (Helios, HPCFDCGL50), and were incubated at 37°C in a humidified atmosphere of 95% air and 5% CO_2_ until HucMSCs slowly crawled out of pieces. After that, the medium was changed every 2-3 day, and HucMSCs underwent passages using Trypin-Express (Gibco, 12604021) solution. HucMSCs were used at passage 4-5 (P4-5) for all experiments.

**Evaluation of HucMSCs phenotype**

The phenotype of HucMSCs mainly included cell surface markers and tri-lineage differentiation. Firstly, after digested and collected, the P4-5 HucMSCs were stained with anti-human antibodies (CD11b/CD19/CD34/CD45/HLA-DR-PE, CD73-APC, CD105-PE, CD90-FITC; 5 μL/each tube) and incubated at 4°C in dark for 30 min. Isotype antibodies served as negative controls. HucMSCs were washed using PBS and centrifuged at 2000 g for 5 min. Secondly, HucMSCs were sorted by FACS Beckman flow cytometer (BD Biosciences, USA), and analysis was performed with Flow Jo software (BD Biosciences, USA). Thirdly 5x10^5^ HucMSCs were seeded and cultured on the 12-well plates by the osteogenic/adipogenic/chondrogenic differentiation medium (Gibco), and then stained by Alizarin Red S, Oil Red O, Toluidine blue, respectively.

**Histological analysis**

After euthanizing animals, heart tissues of MI-N.S and MI-HucMSC groups were fixed in 4% paraformaldehyde (PFA) overnight, embedded in paraffin and sectioned at 3-4 μm intervals. The sections were stained with Hematoxylin and eosin (H&E).

**Immunofluorescence staining**

Firstly, frozen sections of heart tissues were fixed with 4% PFA and blocked with 1% Bovine Serum Albumin (BSA) at 4°C for 30 min. Then, the sections were incubated with anti-CD4 (Abcam, ab183685) and anti-FoxP3 (Abcam, ab215206), at 4°C overnight to visualize immune cells in heart tissue, while 2-(4-Amidinophenyl)-6-indolecarbamidine dihydrochloride (DAPI) was used to mark cell nuclei. After incubated with above antibodies and washed with phosphate buffered saline (PBS), different fields of each slide were randomly selected and photograph under an inverted fluorescent microscope (Leica DM6000B, Germany).

**Flow cytometry analysis**

Briefly, mice were anesthetized with 1% sodium pentobarbital (60mg/kg, intraperitoneal injection), and the heart was exposed and perfused with pre-cold PBS on day 7 after MI. Then, the heart was cut into 1 mm and put into the C-tube with collagenase II solution (1.5 mg/mL, added 500x DNA enzymes). During broken twice using gentleMACS™ Tissue Dissociators (Miltenyi Biotec, USA), the heart tissue was centrifuged at 37°C for 30 min. After removing debris, the supernatant was filtered (70 μm strainer) to obtain single cell suspension. Cells were stained with the following labeled antibodies: anti-mouse CD45 (Biolegend, Clone I3/2.3), CD19 (Biolegend, Clone 1D3/CD19), CD3 (Biolegend, Clone 17A2), CD4 (Biolegend, Clone GK1.5), F4/80 (Biolegend, Clone BM8), Ly-6G (Biolegend, Clone 1A8). Data were acquired on a FACS Beckman flow cytometer (BD Biosciences, USA), and analysis was performed with Flow Jo software (BD Biosciences, USA).

**Cytokine profiling detection**

For cytokine protein expression, the tissue samples of infarct and border area in heart of MI murine were collected and weighed. Then we added 500 μL ProcartaPlex Cell Lysis Buffer (EPX-99999-000) per 100 mg tissue. After tissue homogenization and centrifugation, the protein of sample was quantified by Bicinchoninic Acid (BCA) Protein Assay Kit (Bio-Rad, Hercules, CA, USA) and then detected for cytokine profiling (Thermo Fisher, EPX110-20820-901, EPX01A-20614-901, EPX01A-26005-901 and EPX01A-26009-901).

**Cell migration assay**

The control group collected heart tissues of normal mouse, the MI-N.S and MI-HucMSC groups collected the infarct and border zone of murine hearts. After the protein of cardiac tissues was lysed and quantified by BCA assay, the concentration of C-C Motif Chemokine Ligand 2 (CCL2) was quantified by Elisa assay (absin, abs520016) according to the manufacturer’s instructions. Polycarbonate membrane transwell inserts (24 well, pore size 5.0 μm) were used for CD4^+^ T cell migration assay. At the lower chamber, a total volume of 500 μL RPMI1640 containing 20 μg tissue protein were added. At the upper chamber, a total number 5*10^5^ purified CD4^+^ T cells were added. Sufficient dose CCL2 antibody (R&D system, AF-479-NA) was added to the lower chamber for neutralization with information of CCL2 concentration determined by Elisa assay. Sufficient dose C-C Motif Chemokine receptor 2 (CCR2) antagonist (MCE, HY-108323) was added to the upper chamber with purified CD4^+^ T cells in indicated groups. After co-culturing for 16 h, the CD4^+^ T cells that had migrated to the lower chamber were collected and counted by handheld automated cell counter (millipore).

**Figure legends**

**Sup Fig 1: The phenotype of MSCs from human umbilical cord *in vitro*.** (A) Flow cytometry analyses showed that cell surface markers of HucMSCs were positive for CD73/CD105/CD90 and negative for CD11b/CD19/CD34/CD45/HLA-DR. (B) After induced, HucMSCs were driven to Alizarin Red-positive osteogenic, Oil Red O-positive adipogenic and Toluidine blue-positive chondrogenic differentiation.

**Sup Fig 2: Representative H&E** **immunohistochemical photos of hearts in MI-N.S and MI-HucMSC groups on day 7.** Scale bar=1000 µm.

**Sup Fig 3: Representative immunofluorescence photos of hearts in MI-N.S and MI-HucMSC groups on day 28.** Green, CD4; Red, FoxP3; Blue, DAPI. Scale bar=50 µm.

**Sup Fig 4: Increase of immune cells in the infarcted heart after HucMSCs treatment on day 7.** (A) Representative flow cytometry photos were presented on hearts stained with anti-CD45, F4/80, Ly-6G, CD3, CD19 and CD4 antibody from mice with N.S or HucMSCs. (B) Statistical results of percent of immune cells between MI-N.S and MI-HucMSC groups. **p*<0.05, ***p*<0.01 and ****p*<0.001 versus MI-N.S group. ns, no significant difference versus MI-N.S group.

**Sup Fig 5: Statistical results of the T cells associated cytokines and chemokines in the heart between MI-N.S and MI-HucMSC groups on day 7.** n=12 per group. ns, no significant difference, **p*<0.05 and ***p*<0.01 versus MI-N.S group.

**Sup Fig 6: Neither CCL2 antibody nor CCR2 antagonist have effect on CD4^+^ T cells migration *in vitro*.** ^##^*p*<0.01 versus Control group, ^&^*p*<0.05 versus MI-N.S group, *p*=ns versus MI-HucMSC group.

**Table S1. The primer sequences information.**

**Sup Fig 1**

**
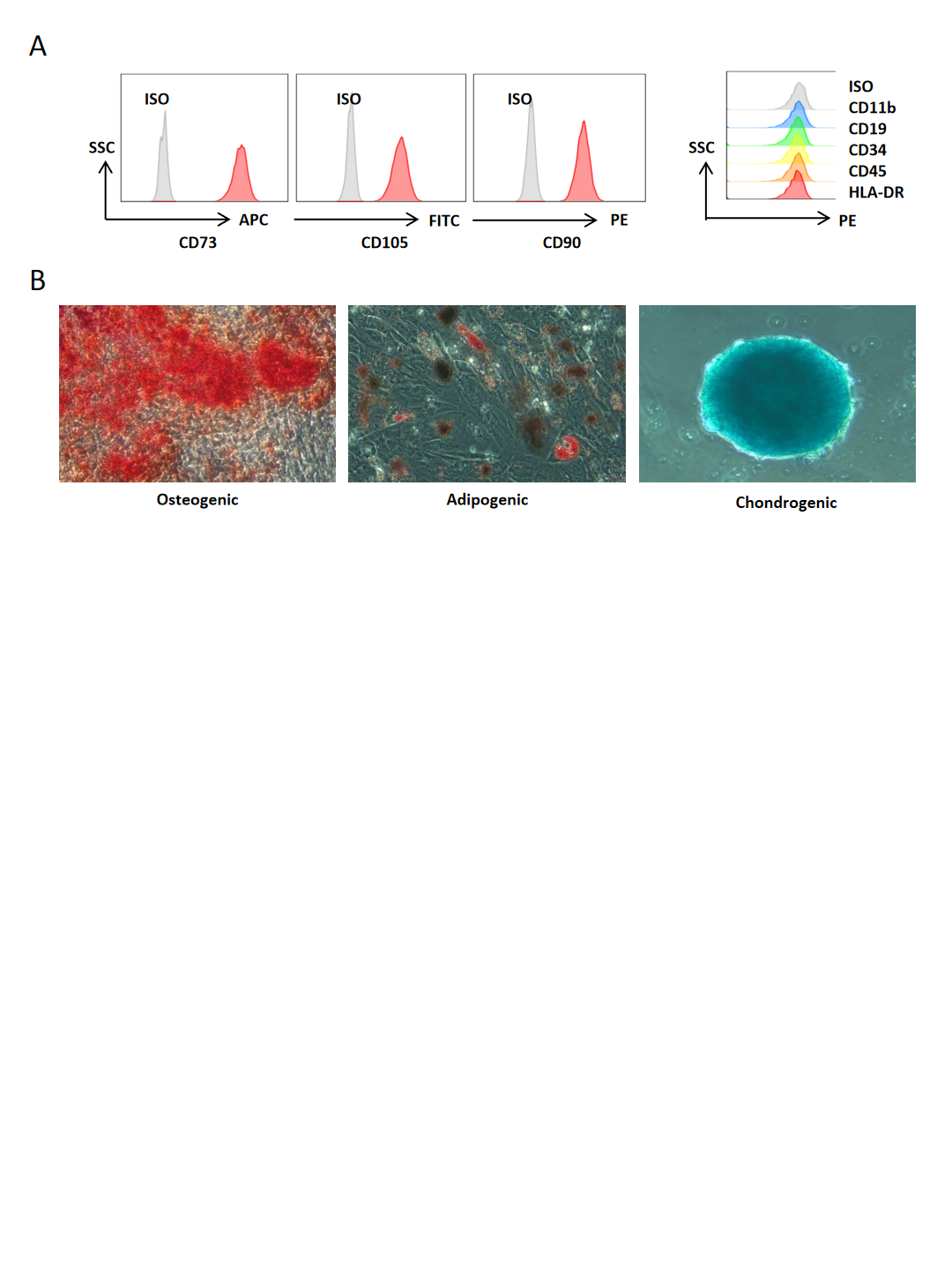
**

**Sup Fig 2**

**
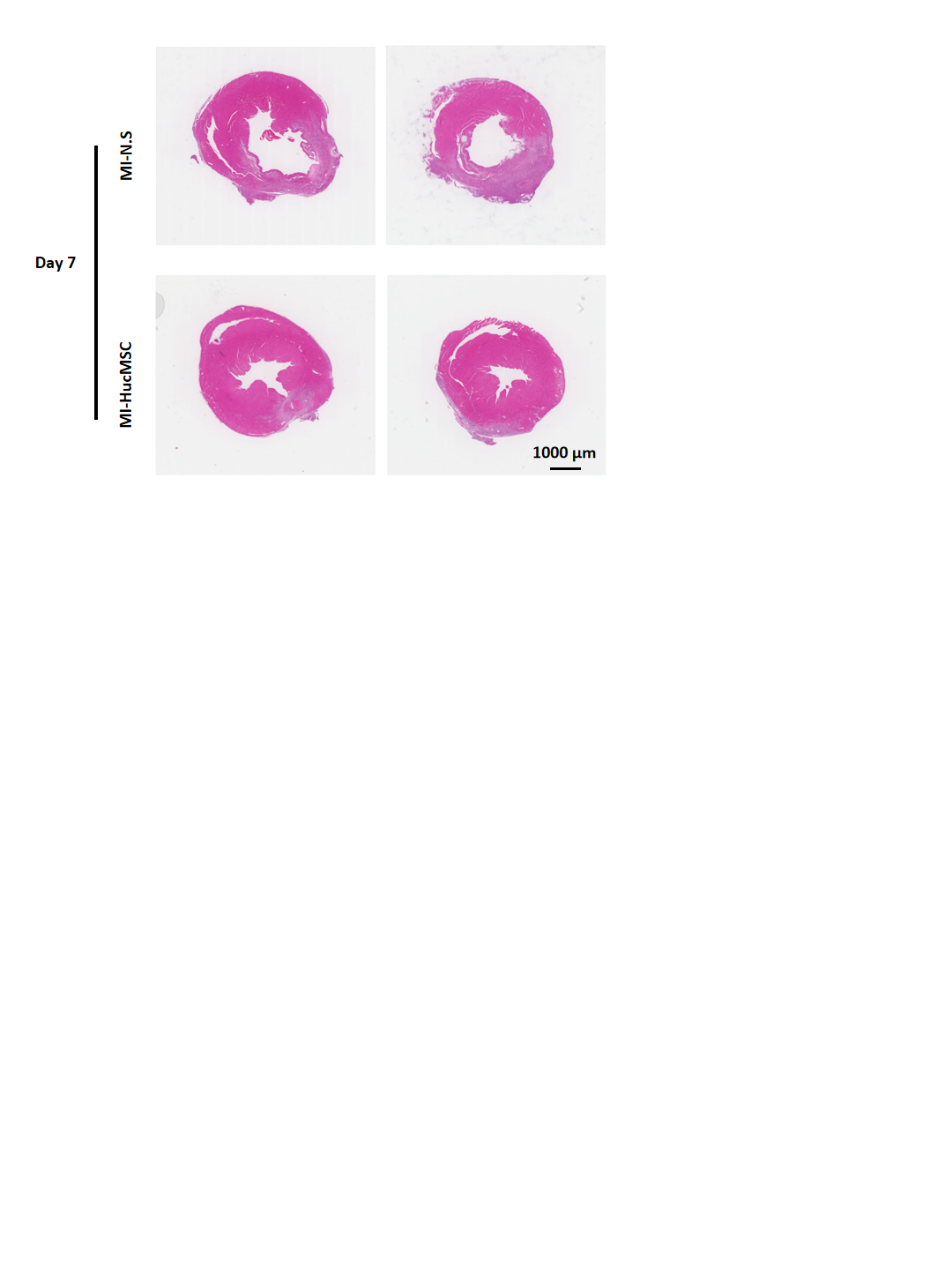
**

**Sup Fig 3**

**
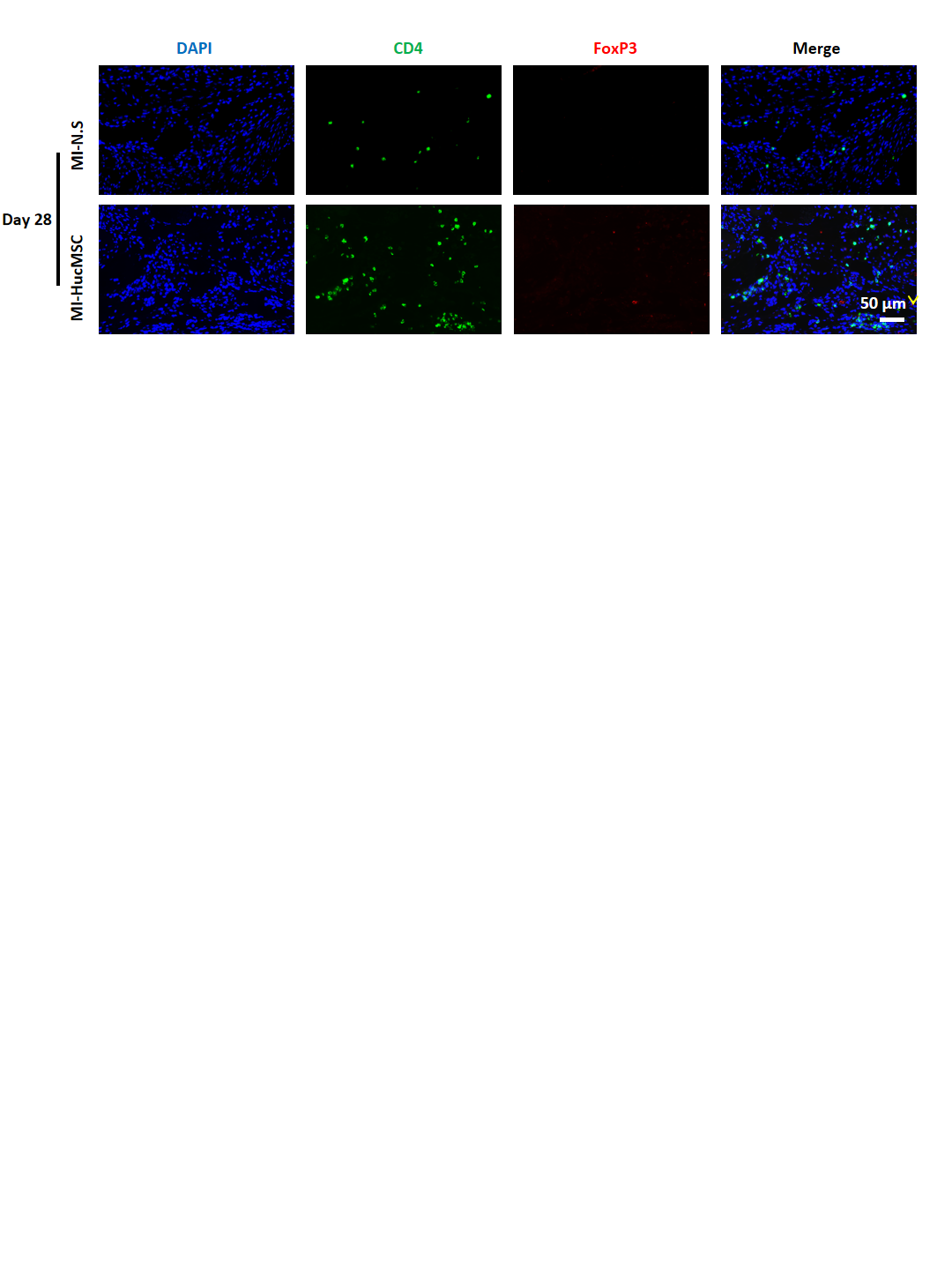
**

**Sup Fig 4**

**
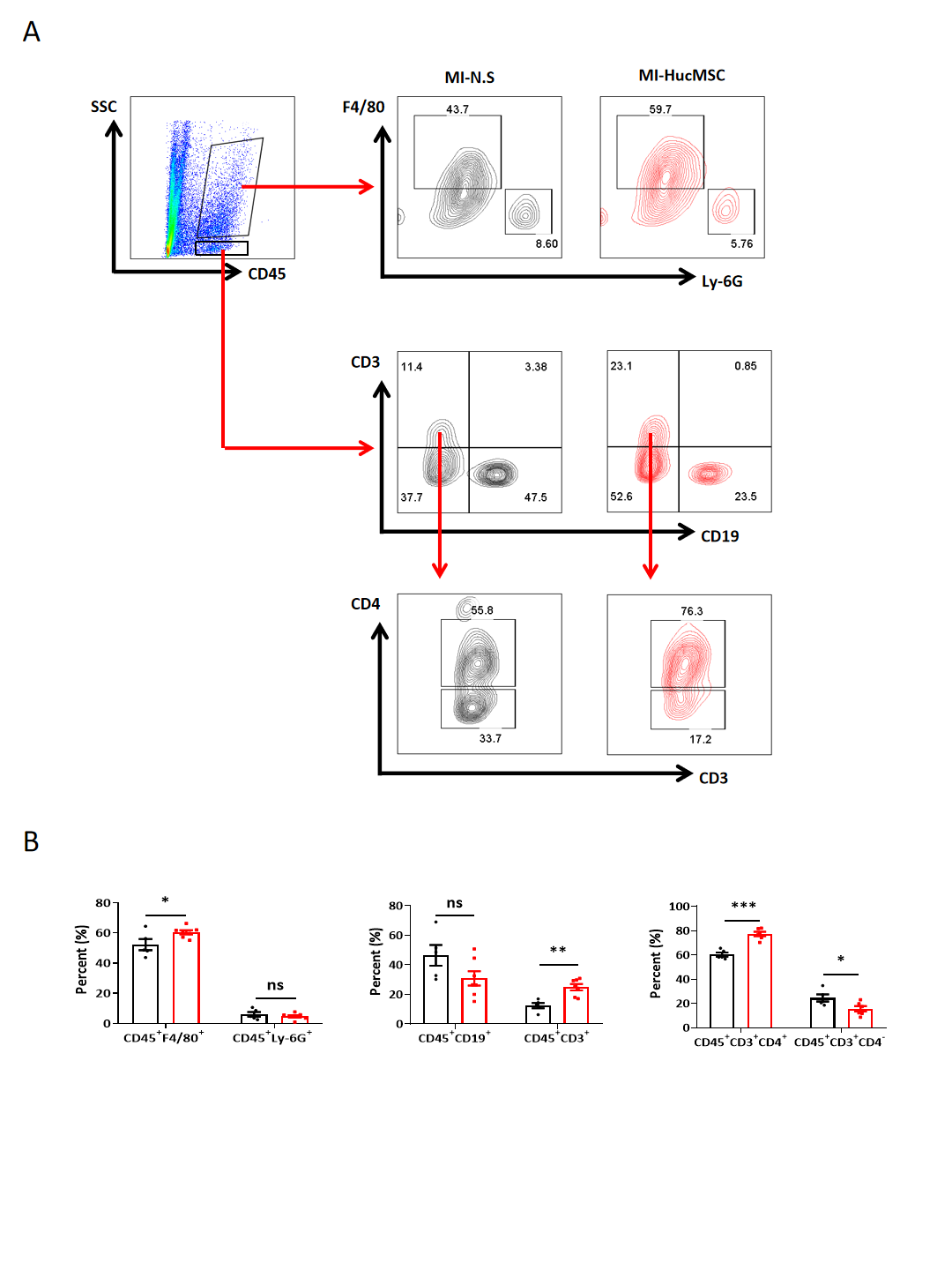
**

**Sup Fig 5**

**
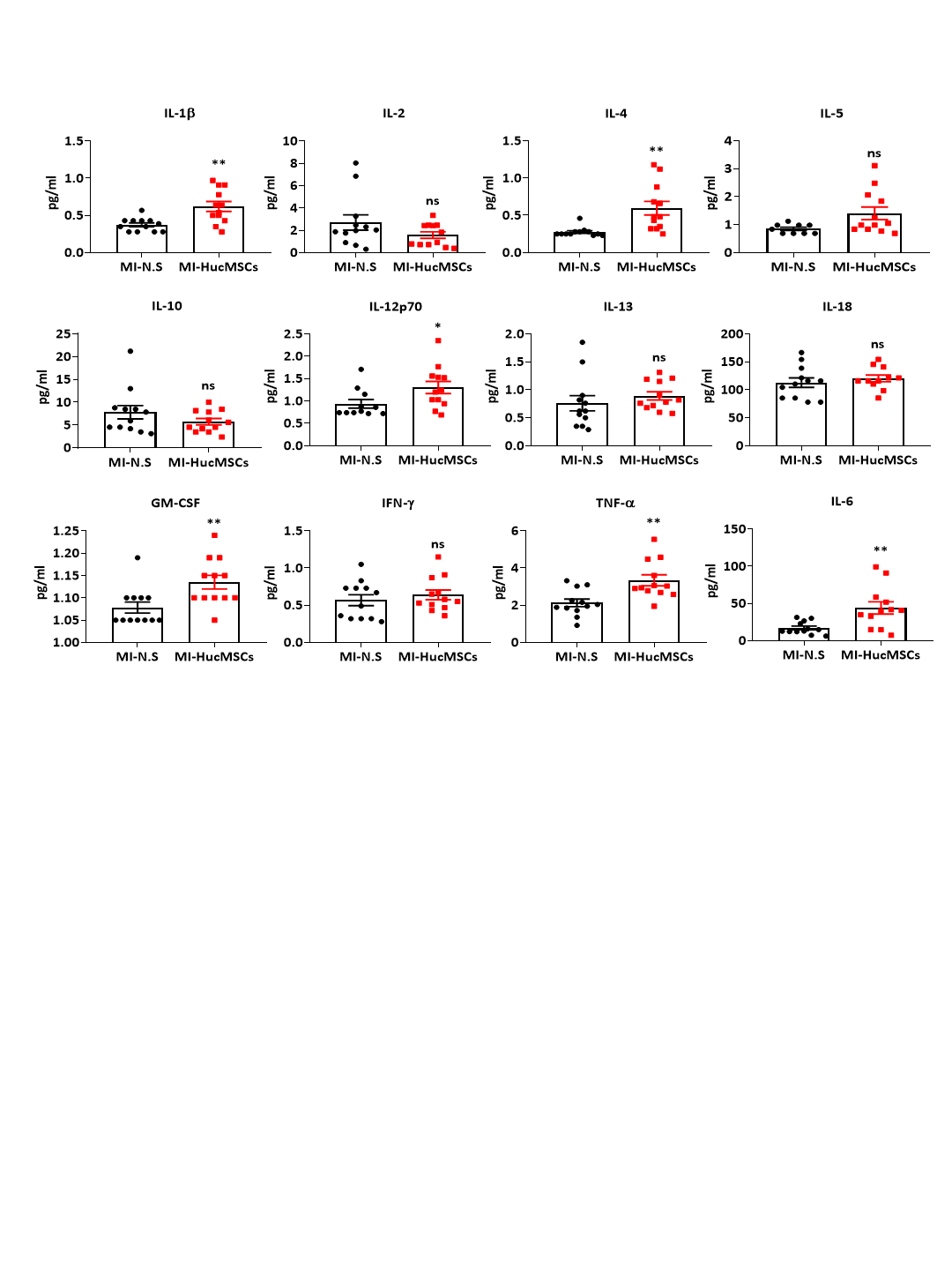
**

**Sup Fig 6**


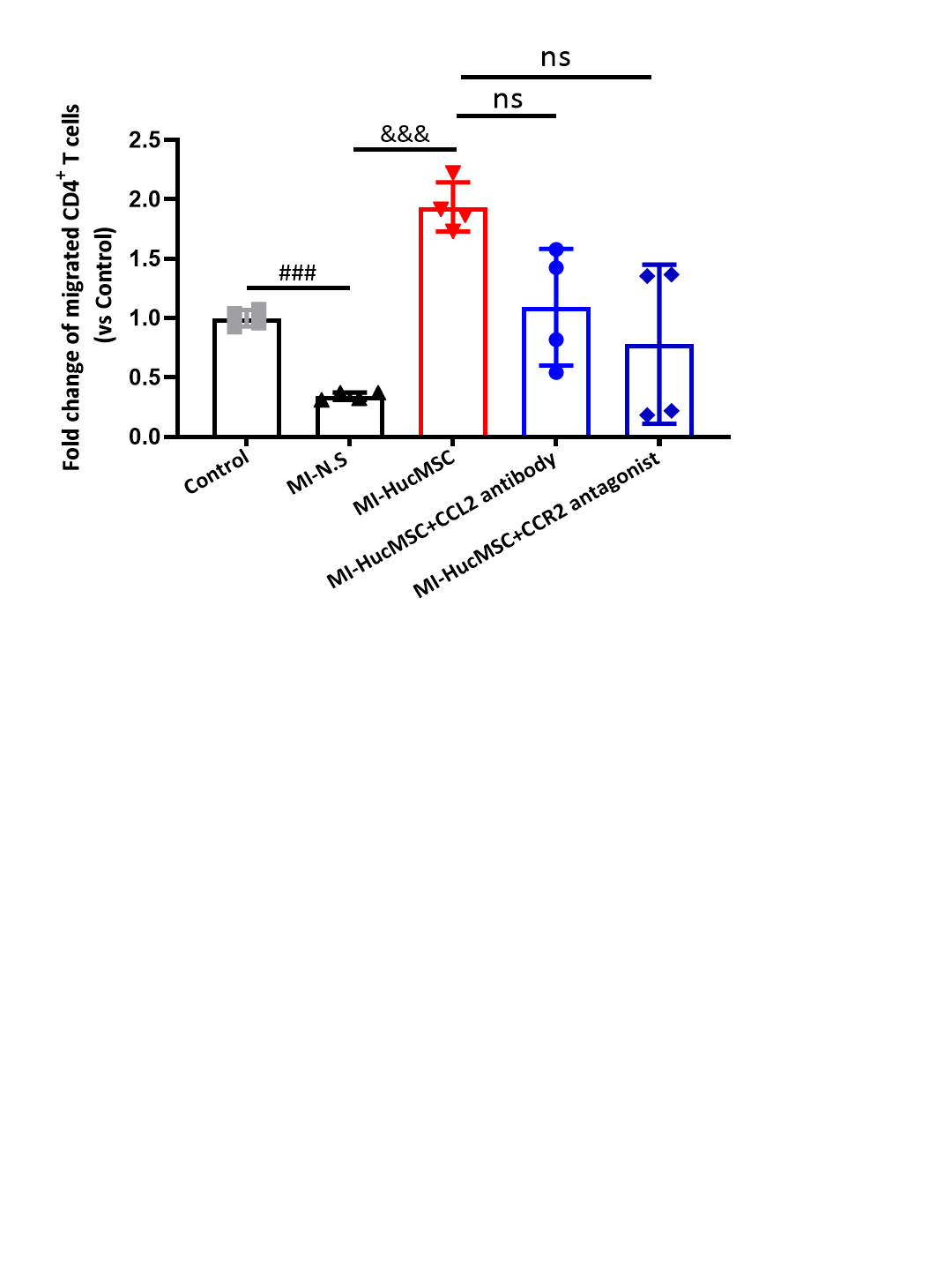


**Table S1. The primer sequences information.**

|  | F | R |
| --- | --- | --- |
| COL I | CCTCAGGGTATTGCTGGACAAC | CAGAAGGACCTTGTTTGCCAGG |
| COL III | GACCAAAAGGTGATGCTGGACAG | CAAGACCTCGTGCTCCAGTTAG |
| MMP2 | CAAGGATGGACTCCTGGCACAT | TACTCGCCATCAGCGTTCCCAT |
| TIMP2 | AGCCAAAGCAGTGAGCGAGAAG | GCCGTGTAGATAAACTCGATGTC |
| ANP | TACAGTGCGGTGTCCAACACAG | TGCTTCCTCAGTCTGCTCACTC |
| BNP | TCCTAGCCAGTCTCCAGAGCAA | GGTCCTTCAAGAGCTGTCTCTG |
| MYH7 | GCTGGAAGATGAGTGCTCAGAG | TCCAAACCAGCCATCTCCTCTG |
| MYH6 | GCTGGAAGATGAGTGCTCAGAG | CCAGCCATCTCCTCTGTTAGGT |
| VEGF-a | CTGCTGTAACGATGAAGCCCTG | GCTGTAGGAAGCTCATCTCTCC |
| GAPDH | CATCACTGCCACCCAGAAGACTG | ATGCCAGTGAGCTTCCCGTTCAG |
